# Somatic Mutation Profiles Revealed by Next Generation Sequencing (NGS) in 39 Chinese Hepatocellular Carcinoma Patients

**DOI:** 10.3389/fmolb.2021.800679

**Published:** 2022-01-18

**Authors:** Lixin Ke, Jianming Shen, Jikun Feng, Jialin Chen, Shunli Shen, Shaoqiang Li, Ming Kuang, Lijian Liang, Cuncun Lu, Dongming Li, Qiang He, Baogang Peng, Yunpeng Hua

**Affiliations:** ^1^ Hepatobiliary and Pancreatic Center, The First Affiliated Hospital, Sun Yat-sen University, Guangzhou, China; ^2^ Sun Yat-sen University Cancer Center, Guangzhou, China; ^3^ Institute of Basic Research in Clinical Medicine, China Academy of Chinese Medical Sciences, Beijing, China

**Keywords:** hepatocellular carcinoma, next generation sequencing, TP53, targeted therapy, SNV, CNV, INDEL

## Abstract

The features and significance of somatic mutation profiles in hepatocellular carcinoma (HCC) have not been completely elucidated to date. In this study, 39 tumor specimens from HCC patients were collected for gene variation analysis by next-generation sequencing (NGS), and a correlation analysis between mutated genes and clinical characteristics was also conducted. The results were compared with genome data from cBioPortal database. Our study found that T > G/A > C transversions (Tv) and C > T/G > A transitions (Ti) were dominant. The sequence variations of TP53, MUC16, MUC12, MUC4 and others, and the copy number variations (CNVs) of FGF3, TERT, and SOX2 were found to be more frequent in our cohort than in cBioPortal datasets, and they were highly enriched in pathways in cancer and participated in complex biological regulatory processes. The TP53 mutation was the key mutation (76.9%, 30/39), and the most common amino acid alteration and mutation types were p.R249S (23.5%) and missense mutation (82.3%) in the TP53 variation. Furthermore, TP53 had more co-mutations with MUC17, NBPF10, and AHNAK2. However, there were no significant differences in clinical characteristics between HCC patients with mutant TP53 and wild-type TP53, and the overall survival rate between treatment *via* precision medication guided by NGS and that *via* empirical medication (logrank *p* = 0.181). Therefore, the role of NGS in the guidance of personalized targeted therapy, solely based on NGS, may be limited. Multi-center, large sample, prospective studies are needed to further verify these results.

## Introduction

Hepatocellular carcinoma (HCC) is now the fourth most common cause of cancer-related deaths worldwide. Approximately 78,000 patients died from HCC in 2018 (Bray et al., 2018; Forner et al., 2018). Recent next-generation sequencing (NGS)-based studies have uncovered the genetic landscape of HCC (Totoki et al., 2014; Schulze et al., 2015; Cancer Genome Atlas Research, 2017), including driver mutations in TP53, CTNNB1, TERT promoter, and other key gene loci. However, how genetic alterations drive the occurrence and development of HCC remains largely unknown.

As a high-throughput sequencing technique, NGS can perform multiple typological analyses on thousands of genes. The main purpose of NGS is to find the main driver gene in patients with advanced cancer and carry out targeted therapy, as well as to try to discover the molecular mutation target of drug resistance (Deng et al., 2019). An increasing number of clinical studies have shown that the analysis of comprehensive characterization of genome changes has clinical benefits for cancer patients (Takeda et al., 2015; Staaf et al., 2019). However, there are still many unknown pathogenic variants waiting to be discovered. Identification of these alterations in cancer patients is the first step toward providing therapeutic targets.

Herein, we characterized differences of the genomic profiles between HCC patients in our cohort and HCC patients in the cBio Cancer Genomics Portal (cBioPortal, http://cBioPortal.org) database using six datasets (MSK, Clin Cancer Res 2018; INSERM, Nat Genet 2015; MSK, PLOS One 2018; AMC, Hepatology 2014; RIKEN, Nat Genet 2012; TCGA, Firehose Legacy) (Gao et al., 2013). We also explored the correlations between high-frequency mutated genes and clinical characteristics of patients, and compared the efficacy between precision medication guided by NGS and empirical medication.

## Methods

### Patients and Tissue Acquisition

A total of 39 HCC samples were collected for targeted panel or whole-exome sequencing between 2014 and 2019 at the First Affiliated Hospital of Sun Yat-sen University. After obtaining the approval of the Ethics Committee, written informed consent was obtained from all patients. The study inclusion criteria were as follows: 1) age at diagnosis was more than 18 years; 2) HCC samples were confirmed by pathological diagnosis; 3) patients underwent hepatectomy as treatment. The exclusion criteria included the following: 1) patients having other types of malignant tumors in addition to HCC; 2) severe organ damage, autoimmune diseases, and mental illness*.* In addition, patients were grouped according to the Barcelona Clinic Liver Cancer (BCLC) staging system (Forner et al., 2018). Tumor pathological grade was based on the Edmondson-Steiner Grading System (Edmondson and Steiner, 1954).

Tumor samples were collected immediately following surgical resection, and then stored in pre-cold RPMI-1640 medium with 5% FBS and 1 × Penicillin/Streptomycin, or in Histidine-Tryptophan-Ketoglutarate tissue preservation solution if the estimated shipping time was longer than 1 h. Formalin-fixed paraffin-embedded (FFPE) sections of surgical tumor samples were also sent for analysis when fresh tumor samples were unavailable. Samples were anonymized for further analysis.

After discharge, patients were seen in the clinic monthly for the first 6 months, and then every 3 months, as described in our previous study (Ke et al., 2020). Telephonic follow-up was also conducted every 6 months. The diagnosis of tumor recurrence was made based on clinical examination, laboratory data, and radiological examinations (such as MRI, CT, and positron emission tomography [PET] scan).

### Targeted Panel Sequencing, Whole-Exome Sequencing and cBioPortal Database Analysis

The panel of targeted deep sequencing comprised 4,557 exons of 365 tumor-associated genes, and 45 introns from 25 genes where frequent gene fusions could be captured in cancer (Supplementary Figure S1). All targeted panel sequencing assays were performed at the 3D Med Clinical Laboratory Co., Ltd. (Shanghai). The detailed method used to perform targeted deep sequencing has been described elsewhere (Feng et al., 2020). All whole-exome sequencing assays were performed at the GenomiCare Medical Laboratory Co., Ltd. (Shanghai). The process of whole-exome sequencing included the following: 1) exome capture, library construction, and sequencing; 2) sequence mapping and somatic variant detection; and 3) detection of copy-number alterations, which have been described in detail elsewhere (Tan et al., 2016; Yang et al., 2019).

We further used the online analysis tool of the cBioPortal database to explore the differences of mutation profiles between our cohort and cBioPortal datasets. The correlations between the high-frequency mutation gene and clinical characteristics were also analyzed.

### Statistical Analysis

Statistical analyses for clinical data and mutation profiles were performed using SPSS Statistical software, version 25.0 (IBM, Chicago, Illinois, United States) and Excel 2019. Unordered categorical variables were analyzed by Fisher’s exact or Chi-Square test, and ordinal or continuous variables were analyzed by non-parametric Mann–Whitney U test. Correlations were analyzed to identify clinical characteristics related to mutation profiles. Mutation frequency of gene = the number of patients with gene mutation/total number of patients ×100%. Overall survival (OS) was defined as the time from the date of surgery until death or last follow-up, and disease-free survival (DFS) was defined as the time from the date of surgery to initial tumor recurrence, metastasis, or death. The last follow-up was conducted in August 2021. The survival analysis was conducted using the Kaplan–Meier method and compared *via* log-rank test. A two-sided value of *p* < 0.05 was considered to be statistically significant.

## Results

### Clinical Characteristics of Patients

In the present study, we enrolled 39 HCC patients with a median age of 47 years (range, 26–70 years) at diagnosis from May 2014 to December 2019 for targeted panel or whole exome sequencing. These patients consisted of 36 males and 3 females; 5 patients had cirrhosis and 29 were HBsAg positive. There were 24 patients (61.5%), 6 patients (15.4%), 8 patients (20.5%), and 1 patient (2.6%) with Edmondson-Steiner grade II, II-II, III, and IV, respectively. Tumor extrahepatic metastasis occurred in seven patients (17.9%, 7/39). According to BCLC staging system, the number of stage A, B, and C patients was 16 (41.0%), 9 (23.1%), and 14 (35.9%), respectively. Portal vein tumor thrombus (PVTT) and microvascular invasion (MVI) were observed in 13 (33.3%) and 17 (43.6%) patients, respectively. The clinical characteristics of HCC patients are shown in Table 1. Detailed information is shown in Supplementary Table S1.

**TABLE 1:** The clinical characteristics of HCC patients.

Variables	Cases (%)
Age, year	
<60	30 (76.9%)
≥60	9 (23.1%)
Sex	
Male	36 (92.3%)
Female	3 (7.7%)
Liver cirrhosis	
no	34 (87.2%)
yes	5 (12.8%)
HBsAg	
Negative	10 (25.6%)
Positive	29 (74.4%)
HBV-DNA, IU/ml	
<100	21 (53.8%)
≥100	18 (46.2%)
Tumor size, cm	
<5 cm	10 (25.6%)
≥5cm, <10 cm	12 (30.8%)
≥10 cm	17 (43.6%)
Tumor number	
single	20 (51.3%)
multiple	19 (48.7%)
Extrahepatic metastasis	
no	32 (82.1%)
yes	7 (17.9%)
PVTT	
no	26 (66.7%)
yes	13 (33.3%)
MVI	
no	22 (56.4%)
yes	17 (43.6%)
AFP, ng/ml	
<200	17 (43.6%)
≥200	22 (56.4%)
BCLC stage	
A	16 (41.0%)
B	9 (23.1%)
C	14 (35.9%)
Edmondson-Steiner grade	
II	24 (61.5%)
II-III	6 (15.4%)
III	8 (20.5%)
IV	1 (2.6%)

HBV: Hepatitis B virus; PVTT: portal vein tumor thrombus; MVI: microvascular invasion; AFP: alpha fetoprotein; BCLC: barcelona clinic liver cancer.

### Overview of Somatic Mutations in HCC Patients

#### Mutation Identification of Targeted Panel Sequencing

In all, 17 patients underwent targeted sequencing and 117 somatic mutations were identified. Of these, 62.4% (73/117) were single nucleotide variants (SNVs), 32.5% (38/117) were copy number variants (CNVs), and 5.1% (6/117) were insertions/deletion variants (INDELs). Among SNVs, 87.7% (64/73) were missense mutations, 8.2% (6/73) were nonsense mutations, and 4.1% (3/73) were intron variants. With regard to mutation taster prediction, 56 gene variants (47.9%) were deleterious and 61 (52.1%) were unknown (Supplementary Table S2). The commonly mutated genes were TP53 (12.0%, 14/117), TSC2 (1.7%, 2/117), RB1 (1.7%, 2/117), EGF (1.7%, 2/117), CTNNB1 (1.7%, 2/117), BRCA2 (1.7%, 2/117), NTRK3 (1.7%, 2/117), LRP1B (1.7%, 2/117), AXIN1 (1.7%, 2/117), IRS2 (1.7%, 2/117), MCL1 (1.7%, 2/117), and MYC (1.7%, 2/117) (Table 2).

**TABLE 2:** Summary of frequent gene variation in HCC detected by targeted sequencing.

Gene	Cases	Variant type	Amino acid or nucleotide alteration	Mutation frequency/copy number	Mutation type	Mutation taster prediction
TP53	1	SNV	p.V157F	47.70%	missense	deleterious
TP53	2	SNV	p.R249S	0.44%	missense	deleterious
TP53	3	SNV	p.E258*	25.40%	nonsense	deleterious
TP53	3	SNV	p.F270V	2.00%	missense	deleterious
TP53	4	SNV	p.E258K	40.30%	missense	deleterious
TP53	5	SNV	p.R158L	75.30%	missense	deleterious
TP53	7	SNV	c.673-2A > T	54.90%	intron_variant	deleterious
TP53	10	SNV	p.R249S	no available	missense	deleterious
TP53	11	SNV	p.R249S	no available	missense	deleterious
TP53	13	SNV	p.R249S	no available	missense	deleterious
TP53	14	SNV	p.L194R	no available	missense	deleterious
TP53	17	INDEL	p.Q136Hfs*34	no available	frameshift mutation	deleterious
TP53	9	SNV	p.V157F	32.00%	missense	deleterious
TP53	6	SNV	p.R174W	no available	missense	unknown
TSC2	2	SNV	p.E1490G	no available	missense	unknown
TSC2	14	INDEL	exon5-exon16 dup	no available	—	unknown
RB1	1	SNV	p.T168A	no available	missense	unknown
RB1	8	INDEL	p.S393Rfs*8	74.90%	frameshift mutation	deleterious
EGF	8	SNV	p.G392R	no available	missense	deleterious
EGF	9	SNV	p.P644S	no available	missense	deleterious
CTNNB1	4	SNV	p.D32N	8.70%	missense	deleterious
CTNNB1	5	SNV	p.S37F	23.60%	missense	deleterious
BRCA2	2	SNV	p.D1898G	no available	missense	deleterious
BRCA2	7	SNV	p.S767C	no available	missense	deleterious
NTRK3	4	SNV	p.V289E	no available	missense	deleterious
NTRK3	16	SNV	p.V550I	no available	missense	unknown
LRP1B	6	SNV	p.D1096N	no available	missense	unknown
LRP1B	7	SNV	p.Y1865N	no available	missense	unknown
AXIN1	6	INDEL	p.H662Mfs*43	21.30%	frameshift mutation	deleterious
AXIN1	12	SNV	p.W444*	66.50%	nonsense	deleterious
IRS2	5	CNV	—	copy number gain (3)	—	deleterious
IRS2	16	CNV	—	copy number gain (3)	—	deleterious
MCL1	8	CNV	—	copy number gain (10)	—	deleterious
MCL1	10	CNV	—	copy number gain	—	deleterious
MYC	11	CNV	—	copy number gain	—	deleterious
MYC	13	CNV	—	copy number gain	—	deleterious

Mutation taster prediction: prediction of the pathogenicity risk of gene variants.

### Somatic Mutations Profiles in HCC Determined *via* Whole-Exome Sequencing

In all, 22 patients underwent whole-exome sequencing. We mapped the sequence reads to the human reference genome and identified a total of 3,383 somatic SNVs, 468 INDELs, and 31 CNVs (Supplementary Table S3). There were a median of 6.42 (range: 3.03–9.10) somatic mutations per mega-base pair (Mb), 0.085% microsatellite instability (MSI) (range: 0.00–33.00%), 1.4% CNV (range: 0.16–19.56%), and 19.37% objective response rate (ORR) (range: 11.27–23.15%) of immunotherapy expectation (Supplementary Table S4).

**FIGURE 1:**
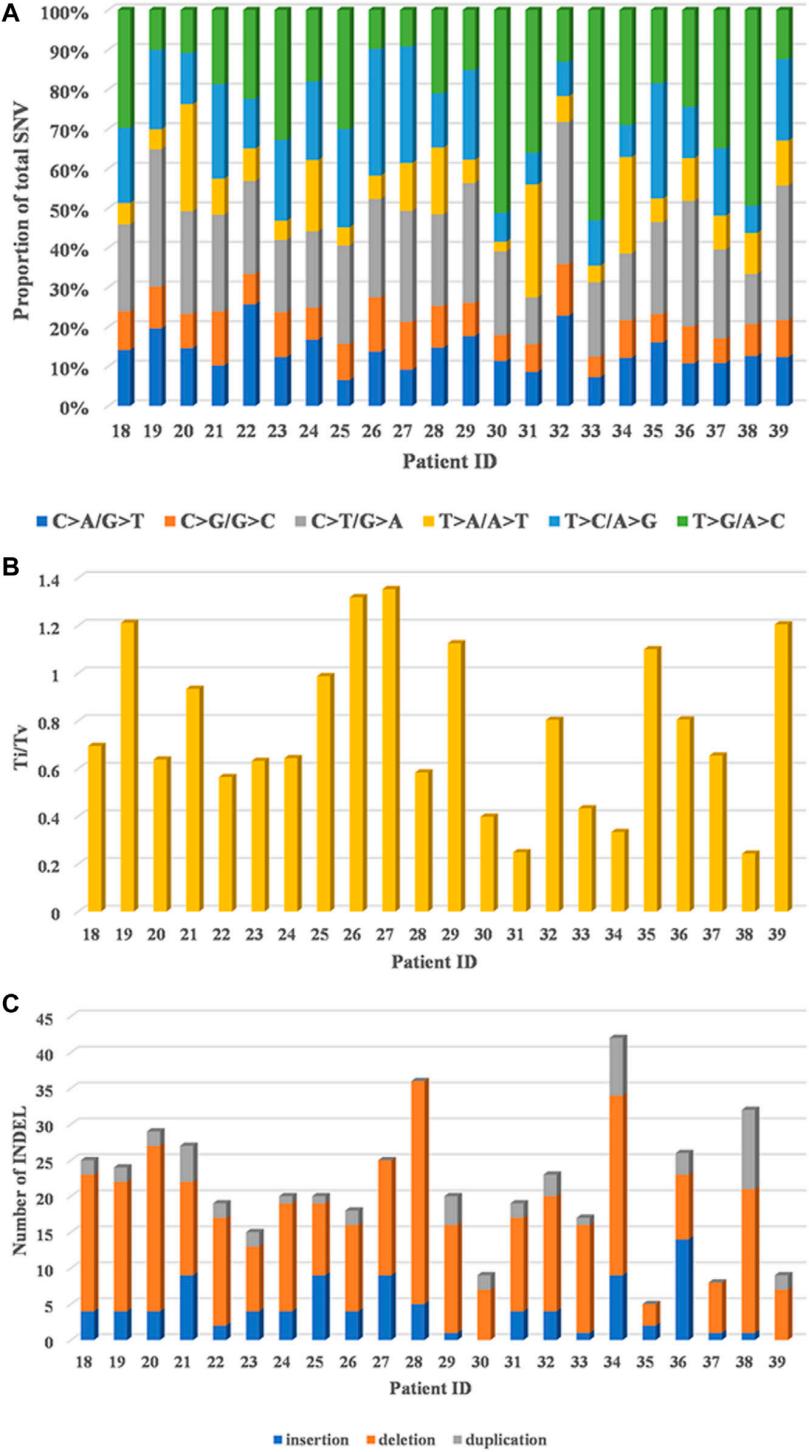
Genomic alterations in 22 HCC patients by whole-exome sequencing. **(A)** Distribution of six substitution patterns. **(B)** The ratio of transition to transversion (Ti/Tv). **(C)** The number of different types of INDELs.

T > G/A > C transversion (Tv) and C > T/G > A transition (Ti) patterns were dominant, C > A/G > T Tv and T > C/A > G Ti were moderate, and the proportion of T > A/A > T Tv and C > G/G > C Tv was the lowest in 22 HCC patients (Figure 1A). In addition, a relatively high ratio of Ti/Tv (median: 0.67; range: 0.24–1.35) was found (Figure 1B). With regard to INDELs, 67.9% (318/468) deletions, 20.3% (95/468) frameshift insertions, and 11.8% (55/468) duplications were observed (Figure 1C).

**FIGURE 2:**
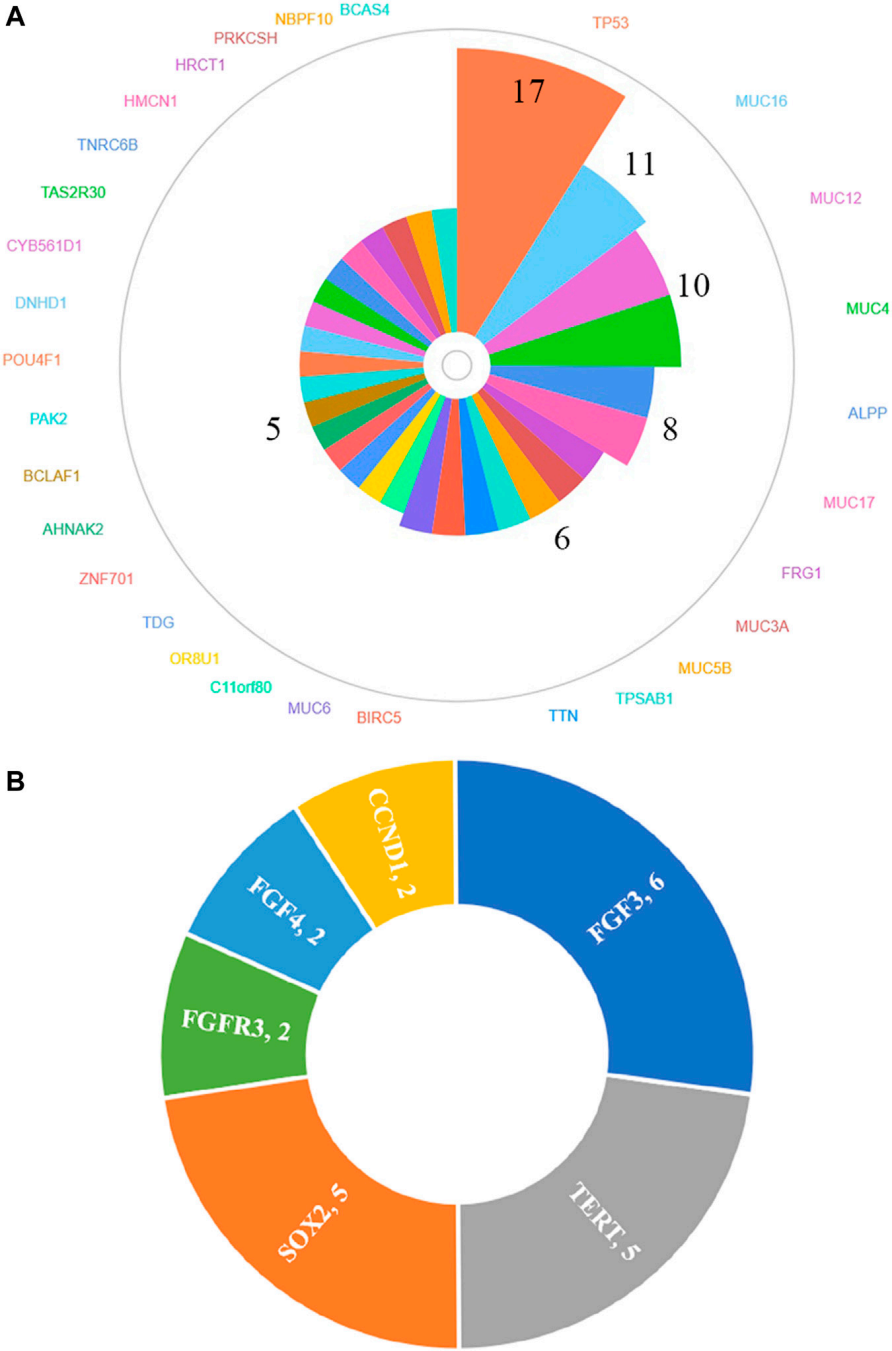
Genes of high-frequency sequence variants **(A)** and CNV **(B)** detected by whole-exome sequencing.

In total, 30 genes, including TP53 (77.3%, 17/22), MUC16 (50.0%, 11/22), MUC12 (45.5%, 10/22), MUC4 (45.5%, 10/22), ALPP (36.4%, 8/22), MUC17 (36.4%, 8/22), FRG1 (27.3%, 6/22), MUC3A (27.3%, 6/22), MUC5B (27.3%, 6/22), TPSAB1 (27.3%, 6/22), TTN (27.3%, 6/22), BIRC5 (27.3%, 6/22), MUC6 (27.3%, 6/22), C11orf80 (22.7%, 5/22), OR8U1 (22.7%, 5/22), TDG (22.7%, 5/22), ZNF701 (22.7%, 5/22), AHNAK2 (22.7%, 5/22), BCLAF1 (22.7%, 5/22), PAK2 (22.7%, 5/22), POU4F1 (22.7%, 5/22), DNHD1 (22.7%, 5/22), CYB561D1 (22.7%, 5/22), TAS2R30 (22.7%, 5/22), TNRC6B (22.7%, 5/22), HMCN1 (22.7%, 5/22), HRCT1 (22.7%, 5/22), PRKCSH (22.7%, 5/22), NBPF10 (22.7%, 5/22) and BCAS4 (22.7%, 5/22), were found to be mutated in at least 20% (5/22) HCC patients by whole-exome sequencing (Figure 2A) and details regarding the top four genes are listed in Supplementary Table S5. We also identified 27 amplified segments, which harbored several known oncogenes such as FGF3, SOX2, and TERT, *etc.* (Figure 2B); and four lost segments, which harbored tumor suppressors including BRCA1, BRCA2, APC, and B2M (Supplementary Table S4).

**FIGURE 3:**
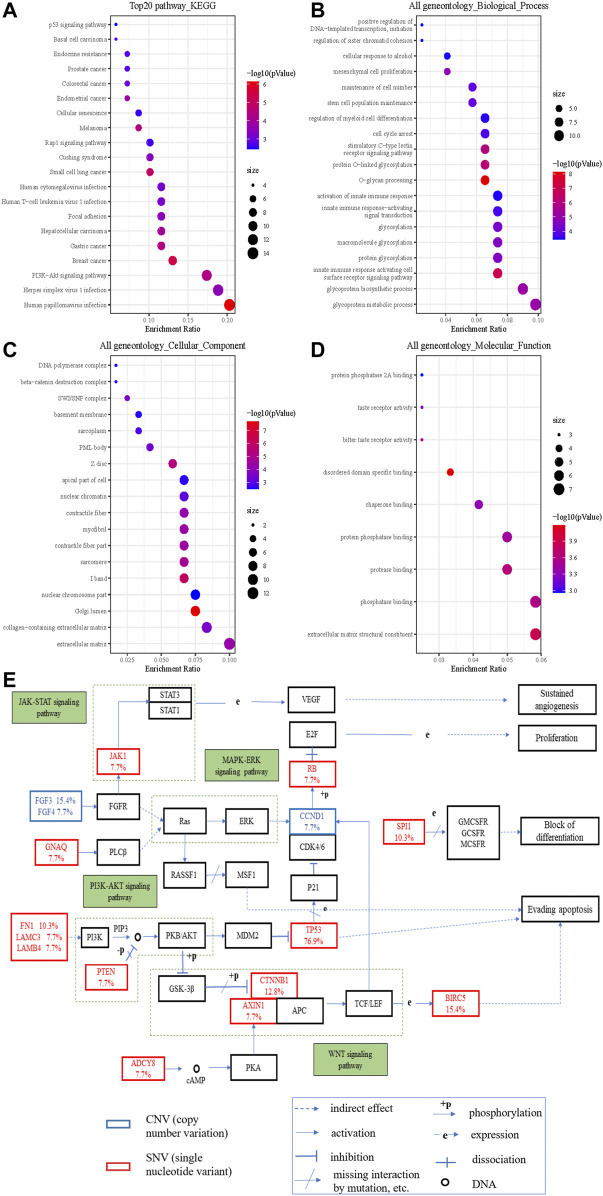
The significantly enriched GO annotations and the KEGG pathways of somatic cell variants in HCC cases. **(A)** KEGG pathway analysis; **(B)** biological processes; **(C)** cellular components; **(D)** molecular functions; **(E)** KEGG pathway annotations of the cancer related pathway, with red lettering denoting SNVs and blue lettering denoting CNVs. The number represents the frequency of variations.

To understand the biological characteristics of the mutated genes, we performed enrichment analysis, which included Gene Ontology (GO) function and Kyoto Encyclopedia of Genes and Genomes (KEGG) pathway analysis. KEGG items revealed that mutated genes were highly enriched in multiple cancer pathways (Figure 3A). With regard to HCC, the cancer we focused on, its enrichment ratio was 11.59% (q = 0.002). GO items demonstrated that mutated genes were mainly involved in glycoprotein metabolic and biosynthetic processes in biological processes (Figure 3B); extracellular matrix and Golgi lumen in cellular components (Figure 3C); and extracellular matrix structural constituents and phosphatase binding in molecular functions (Figure 3D). Figure 3E shows that some variant genes were related to complex cancer pathways. These genes were mainly involved in the JAK/STAT, PT3K/AKT, WNT, and MAPK/ERK pathways, and could influence each other (e.g., in terms of activation, inhibition, and phosphorylation), which could lead to cell evading apoptosis, cell proliferation, sustained angiogenesis, *etc.* and in turn affect the occurrence and development of cancers.

### Variant Types of Key Mutations and Recommendations of Precision Medicine

By targeted panel and whole-exome sequencing, we identified 3,999 somatic variations among 86.4% (3,456/3,999) SNVs, 11.9% (474/3,999) INDELs, and 1.7% (69/3,999) CNVs in 39 HCC patients. It was worth noting that the variation rate of TP53 was the highest by both targeted and whole-exome sequencing (76.5%, 13/17 and 77.3%, 17/22, respectively). The mutation types and mutation taster prediction of TP53 are listed in Table 3. p.R249S was the most common amino acid alteration (23.5%), and 82.3% (28/34) of TP53 variations were missense mutations. Except for p.R174W in case 6, all remaining TP53 variations were deleterious. Furthermore, TP53 was frequently mutated with MUC17 (15.4%, 6/39), NBPF10 (12.8%, 5/39), and AHNAK2 (12.8%, 5/39).

**TABLE 3:** The variant types and mutation taster prediction of TP53.

Type		n (%)
Amino acid or nucleotide alteration		
	p.R249S	8 (23.5%)
	p.V157F	2 (5.9%)
	p.C176Y	1 (2.9%)
	p.C176W	1 (2.9%)
	p.R249W	1 (2.9%)
	p.H179Y	1 (2.9%)
	p.G226fs	1 (2.9%)
	p.H178P	1 (2.9%)
	p.R337L	1 (2.9%)
	p.S215G	1 (2.9%)
	p.R273C	1 (2.9%)
	p.R337C	1 (2.9%)
	p.R273H	1 (2.9%)
	p.G105S	1 (2.9%)
	p.P151S	1 (2.9%)
	p.R213*	1 (2.9%)
	p.G105V	1 (2.9%)
	p.E258*	1 (2.9%)
	p.F270V	1 (2.9%)
	p.E258K	1 (2.9%)
	p.R158L	1 (2.9%)
	c.673-2A > T	1 (2.9%)
	p.L194R	1 (2.9%)
	p.Q136Hfs*34	1 (2.9%)
	p.G245D	1 (2.9%)
	p.R174W	1 (2.9%)
Mutation type		
	missense	28 (82.3%)
	frameshift	2 (5.9%)
	nonsense	2 (5.9%)
	other	2 (5.9%)
Mutation taster prediction		
	deleterious	33 (97.1%)
	unknown	1 (2.9%)

According to the data obtained from targeted panel and whole-exome sequencing reports, 59.0% (23/39) patients had at least one clinically actionable somatic mutation for which clinical treatments could be prescribed using precision medicine (Supplementary Tables S2, S4).

### The Differences of Genomic Profiles Compared With cBioPortal Datasets

Because 30 mutated genes and 3 amplified genes variated in at least 20% HCC patients, we further used cBioPortal database to explore the differences between our cohort and other cohorts. Mutated and amplified genes mentioned above were found in 594 cases and 71 cases, respectively. The common gene variation frequencies were as follows: TP53 (29%), TTN (23%), MUC16 (14%), HMCN1 (7%), MUC4 (6%), and AHNAK2 (5%). The gene variation frequencies for all other genes were less than 5% (Supplementary Figure S2). We found that except for TTN (15% vs. 23%), the mutation frequencies of most commonly mutated genes in our cohort all higher than those in cBioPortal: TP53 (77.3% vs. 29.0%), MUC16 (50.0% vs. 14.0%), MUC12 (45.5% vs. 1.8%), MUC4 (45.5% vs. 6.0%), ALPP (36.4% vs. 0.7%), MUC17 (36.4% vs. 4.0%), FRG1 (27.3% vs. 0.3%), MUC3A (27.3% vs. 0.1%), MUC5B (27.3% vs. 3.0%), TPSAB1 (27.3%, 0.2%), BIRC5 (27.3% vs. 0.1%), MUC6 (27.3% vs. 1.9%), C11orf80 (22.7% vs. 0.3%), OR8U1 (22.7% vs. 0.3%), TDG (22.7% vs. 0.6%), ZNF701 (22.7% vs. 0.7%), AHNAK2 (22.7% vs. 5%), BCLAF1 (22.7% vs. 2.1%), PAK2 (22.7% vs. 0.6%), POU4F1 (22.7% vs. 0.6%), DNHD1 (22.7% vs. 2.4%), CYB561D1 (22.7% vs. 0.1%), TAS2R30 (22.7% vs. 0.3%), TNRC6B (22.7% vs. 1.4%), HMCN1 (22.7% vs. 7%), HRCT1 (22.7% vs. 0.1%), PRKCSH (22.7% vs. 0.5%), NBPF10 (22.7% vs. 2.2%) and BCAS4 (22.7% vs. 0.1%). As for CNVs, the FGF3 amplification rate of 15.4% (6/39), TERT amplification rate of 12.8% (5/39), and SOX2 amplification rate of 12.8% (5/39) in our cohort (Figure 2B) were also significantly higher than those found in the cBioPortal datasets (5.0, 4.0, and 1.1%, respectively, Supplementary Figure S3).

We further used cBioPortal to analyze the variated types of top 4 mutated genes in our cohort. We found 209 missense mutations, 93 truncating mutations, and 5 in-frame mutations in TP53; 136 missense mutations, 15 truncating mutations, and 1 in-frame mutation in MUC16; 15 missense mutations and 2 truncating mutations in MUC12; and 50 missense mutations, 2 truncating mutations, and 2 in-frame mutations in MUC4 (Supplementary Figure S4, Supplementary Table S6). This was similar to our results shown in Supplementary Table S5 indicating that the top 4 mutated genes were dominated by missense and truncating mutations.

### Correlation Analyses Between Gene Mutation and Clinical Characteristics

We used cBioPortal HCC cohorts to analyze the correlations between TP53 mutation and clinical characteristics (Supplementary Table S7) and found that only neoplasm histologic grade (q = 0.008) and race category (q = 0.003) had a significant association with TP53 mutation. With regard to OS and DFS, the survival differences between the TP53 mutation group and the wild-type group were significant in the cBioPortal dataset (OS: logrank *p* = 0.018; DFS: logrank *p* = 0.005) (Supplementary Figure S5). However, the results were different from our study which showed that there were no significant differences in survival outcomes (OS, logrank *p* = 0.084; DFS, logrank *p* = 0.201) as well as other clinical characteristics between the TP53 mutation group and the wild-type group. Cirrhosis tended to occur in FGF3 and MUC4 mutation groups (*p* = 0.019 and 0.011, respectively) (Table 4).

**TABLE 4:** Correlations among FGF3 mutation, MUC4 mutation and cirrhosis.

		FGF3		*p* value	MUC4		*p* value
		Wild type	Mutation		Wild type	Mutation	
Cirrhosis	no	31 (91.2%)	3 (8.8%)	0.019	28 (82.4%)	6 (17.6%)	0.011
	yes	2 (40.0%)	3 (60.0%)		1 (20.0%)	4 (80.0%)	

**FIGURE 4:**
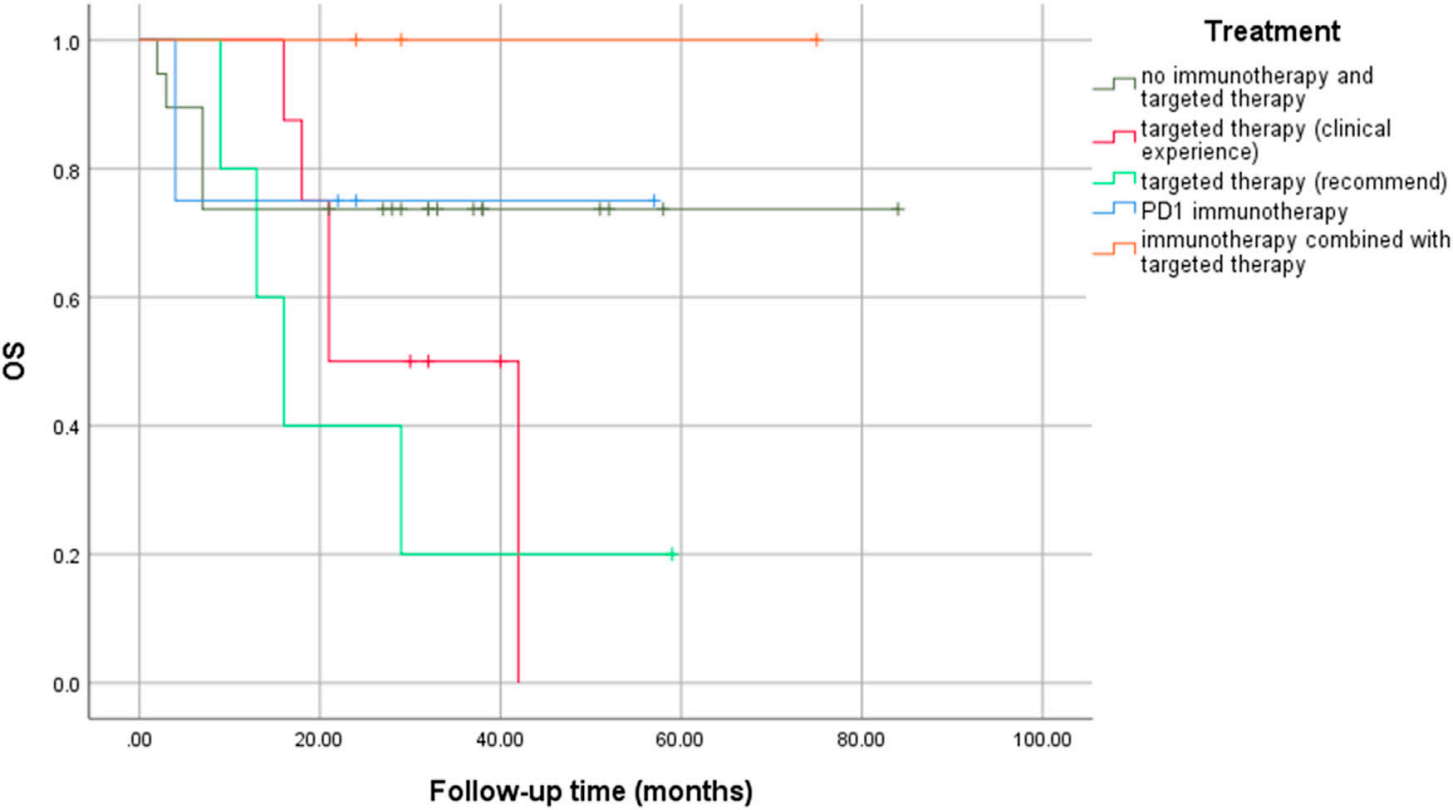
Comparisons of OS rates in different treatment groups.

No significant statistical differences were observed between precision medication guided by NGS and empirical medication (logrank *p* = 0.181), especially between targeted therapy based on recommended drugs and clinical experience (logrank *p* = 0.376) (Figure 4). However, immunotherapy combined with targeted therapy seemed to result in a longer OS rate, even if there was no statistical difference.

## Discussion

In this study, we used NGS to detect multi-gene variations in HCC patients, analyzed the correlations with clinical characteristics, and compared our findings with those of the cBioPortal database.

First, we described the overall situation of somatic mutations. C > A/G > T Tv and T > C/A > G Ti were moderate in our study, and were shared by other HCC cohorts (Totoki et al., 2014; Schulze et al., 2015; Fujimoto et al., 2016). T > G/A > C Tv and C> T/G > A Ti patterns were dominant, but the proportion of T > A/A > T Tv and C > G/G > C Tv was the lowest, implying that T > G/A > C Tv and C > T/G > A Ti may have contributed to hypermutations in our cohort, but these results were different from two previous studies (where T > A/A > T Tv was dominant) (Gao et al., 2019; Zhou et al., 2019) and the Cancer Genome Atlas (TCGA) dataset (where T > G/A > C Tv showed the lowest occurrence) (Supplementary Figure S6), which may be the reason of the complexity of the genome, individual differences and small sample size. In addition, a relatively high ratio of Ti/Tv was found, in agreement with the results of previous HCC sequencing studies (Guichard et al., 2012; Huang et al., 2012) and other cancers studies (Moore et al., 2003; Hainaut and Pfeifer, 2016). Therefore, a high ratio of Ti/Tv in our study may have contributed to the biochemical structure of nucleotides and the chemical characteristics of complementary base pairing (Taylor et al., 2006; Massey, 2015; Stoltzfus and Norris, 2016), which could help researchers gain a deeper understanding of the patterns and strengths of molecular system development and HCC evolution.

Second, we analyzed the main somatic gene variations and found that most of high-frequency mutations in our cohort were relatively low-frequency mutations in cBioPortal datasets. The top 4 mutated genes (TP53, MUC16, MUC12, and MUC4) were dominated by missense mutations, which was similar to the cBioPortal data. Further, TP53 mutations were the most frequent mutation in both our cohort (p.R249S was the most common amino acid alteration) and cBioPortal datasets, even if there was a significant difference in the mutation rate (76.9% vs 29.0%, respectively). In addition, the most common changes in CNV were FGF3, TERT, and SOX2, and their variant rates were all higher than those reported in cBioPortal-HCC patients (15.4% vs. 5.0%; 12.8% vs. 4.0%; 12.8% vs. 1.1%, respectively). We speculated that because of ethnic and individual differences, the genetic profile characteristics of HCC patients in China may be different from those in other countries (three datasets from the United States, one dataset from Europe, one dataset from Korea, the other dataset from Japan in cBioPortal database). Accordingly, large cohort studies are needed to verify these results.

We further explored whether mutant genes were related to clinical characteristics. TP53 mutation had significant correlations with histological grade, race category, OS, and DFS in cBioPortal database. However, our results suggested that there were little correlations between gene variations and clinical characteristics except that cirrhosis tended to occur in FGF3 and MUC4 mutation groups. We further found that the effect of treatments guided by NGS may be limited. There may be several reasons for this difference. First, individual differences, racial disparities, and sample sizes could have affected the results. Next, gene mutations (e.g., nonsense mutation) may not affect the protein expressions, which play a significant role in performing life functions. Further, co-occurring genetic alterations could alter the biological characteristics of tumors and affect the prognosis of patients (Deng et al., 2019), meaning that different genetic mutations may affect each other. Furthermore, enrichment analysis showed that the mutated genes were involved in complex cancer signaling pathways (e.g., PI3K/AKT, WNT, and JAK/STAT pathways), biological processes (e.g., glycoprotein metabolic process, protein glycosylation, and activation of innate immune response), cellular components (e.g., extracellular matrix, Golgi lumen, and nuclear chromosome part), and molecular functions (e.g., extracellular matrix structural constituent, phosphatase binding, and protease binding). When targeted drugs act on HCC cells, tumor cells can change the expression of related proteins, adjust the connection of signal pathways, and change the microenvironment to evade targeted drug attacks. When a pathway is inhibited by targeted drugs, HCC cells can strengthen the signal transduction of other pathways by compensation, thereby re-promoting its own proliferation and invasion, leading to the failure of targeted therapy (Mir et al., 2017). Some targeted drugs can inhibit the angiogenesis of HCC tissues, but a continuous anti-angiogenesis effect can cause tumor starvation and hypoxia, promoting the proliferation of resistant HCC cells that adapt to hypoxia and lack of nutrients (Mendez-Blanco et al., 2018). Thus, intervention of a signaling pathway alone may be ineffective, and the negative feedback may result in the development of drug resistance. Accordingly, the use of several molecularly targeted agents in combination is an appealing way to counteract resistance. Finally, insignificant statistical differences may also be caused by the relatively small sample size. Multi-center, large sample, prospective studies are needed to further verify these results.

No therapeutic targets in many patients suggested that HCC is not completely caused by mutations, or that there are no approved drugs targeting these mutations. Moreover, targeted drugs may be invalid. SHIVA, a randomized trial conducted in France, found that there were no differences between NGS-guided treatment and conventional treatment in terms of PFS and OS (Le Tourneau et al., 2015). In addition, tumor mutation burden (TMB) can also fail to predict immune checkpoint blockade response (McGrail et al., 2018; McGrail et al., 2021). Therefore, the out-of-range use of NGS for targeted drugs should be focused on.

Further, the results of gene sequencing may vary considerably. For instance, different institutions may provide different results for gene sequencing, which may result from discrepancies in sequencing principles, sequencing systems, and bioinformatics algorithms, *etc.* Problems in the gene sequencing process (such as hardware, software, samples, and quality control) can also lead to false negatives or false positives (Xuan et al., 2013; Bean et al., 2020). Moreover, the different understandings of genes or treatments with potential clinical benefits may lead to different interpretations of the same test results (Rehm et al., 2013). Most institutions only rely on public databases to interpret data and recommend targeted drugs, but they fail to conduct individualized analysis based on patient-specific conditions. Therefore, some treatments, which are based on clinical experience rather than gene sequencing, may also be effective. This phenomenon can explain why precision medication guided by NGS was not superior to empirical medication in terms of OS rate in our study. It is worth discussing whether better the results can be obtained with more gene sequencing. If gene sequencing can only help a small number of patients, the incremental cost will be high when it is promoted. In addition, the results of gene sequencing could be useless for treatment if they are not sufficiently correlated with important clinical data (such as tumor size, family history, and drug use). Therapies only based on some gene signaling pathway theories and little literature evidence alone will hardly have any positive effects.

Therefore, gene sequencing may not be translated into improved patient outcomes and the detection of therapeutic gene mutations could be far from having a true clinical benefit. Some studies have reported that patients achieved good curative effects by implementing targeted therapy based on gene sequencing, but the sample size, methodology, and research design were not rigorous and the effective rate was also not mentioned in these studies (Yu et al., 2018; Sun et al., 2020). The effective rate of even programmed death 1 (PD-1) treatment was only 17%–20% (El-Khoueiry et al., 2017; Zhu et al., 2018; Lee et al., 2020). Nevertheless, targeted therapy-combined immunotherapy could improve efficacy, not only in our results but also in other studies (Finn et al., 2020; Xu et al., 2021). Therefore, different patients should choose different gene sequencing based on individual differences and genetic polymorphisms. Molecular biology experts, pathologists, oncologists, bioinformatics experts, and immunology experts should work together to find the best-matched therapeutic drugs and conduct cutting-edge clinical trials for each mutation site so as to provide a comprehensive interpretation of the genetic sequencing report for cancer patients. At the same time, researchers should perform reasonable clinical research, strictly define the outcome of clinical benefit, and prospectively evaluate the efficacy of targeted drugs under the guidance of gene sequencing.

Our study has several strengths. First, we described the somatic mutations profiles and identified the high-frequency variated genes in 39 Chinese HCC patients. Second, similarities and differences were revealed between our HCC cohort and cBioPortal-HCC patients with regard to genomic profiling, especially those genes that were relatively low-frequency in the cBioPortal database but commonly mutated in our cohort. Third, the correlations between gene mutation and clinical characteristics were also analyzed, and its limited values for guiding the clinical work were indicated. However, there are several limitations to our study. First, the sample size of the group was small. Accordingly, large umbrella trials of personalized precision therapy are needed to confirm our findings. Second, we did not perform multiple sequencing methods (such as transcriptomics, proteomics, and metabolomics), cell- and animal-based experiments to further verify the results. Third, the combination of the two sequencing methods may be confusing. For the reason of timeliness, we initially used targeted panel sequencing, and later adopted whole-exome sequencing for a larger genome screen. We wanted to expand the sample size so that the data can be fully utilized. In addition, samples are also being accumulated in our center to further verify our research results. Despite these limitations, this study reflected real-world clinical practice as it related to personalized targeted therapy guided by NGS in patients.

In conclusion, the characteristic somatic mutation profiles in 39 Chinese HCC patients were described in this study. Further, we conclude that the role of NGS in guiding treatment may be limited.

## Data Availability

The data presented in the study are included in the article/Supplementary Material and deposited in the NCBI repository, accession number PRJNA787229.
