# Supplementary figures and images for: Somatic Mutation Profiles Revealed by Next Generation Sequencing (NGS) in 39 Chinese Hepatocellular Carcinoma Patients
Source: Front Mol Biosci. 2022 Jan 18;8:800679. doi: 10.3389/fmolb.2021.800679 (PMC8804344; doi:10.3389/fmolb.2021.800679)

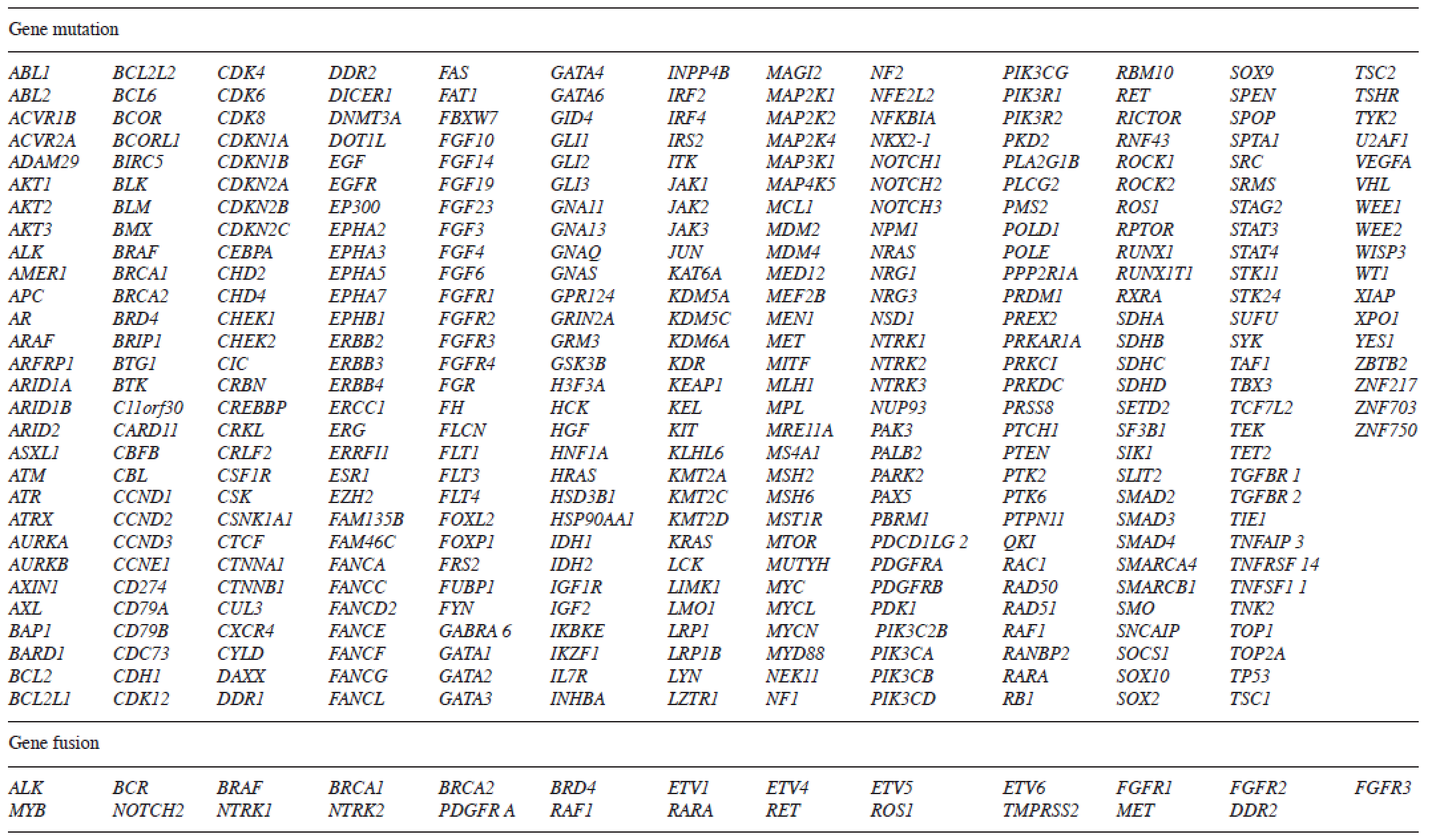

Supplement: Supplementary file 3 [file DataSheet2.ZIP › Supplementary Figures/Supplementary Figure 1.png]

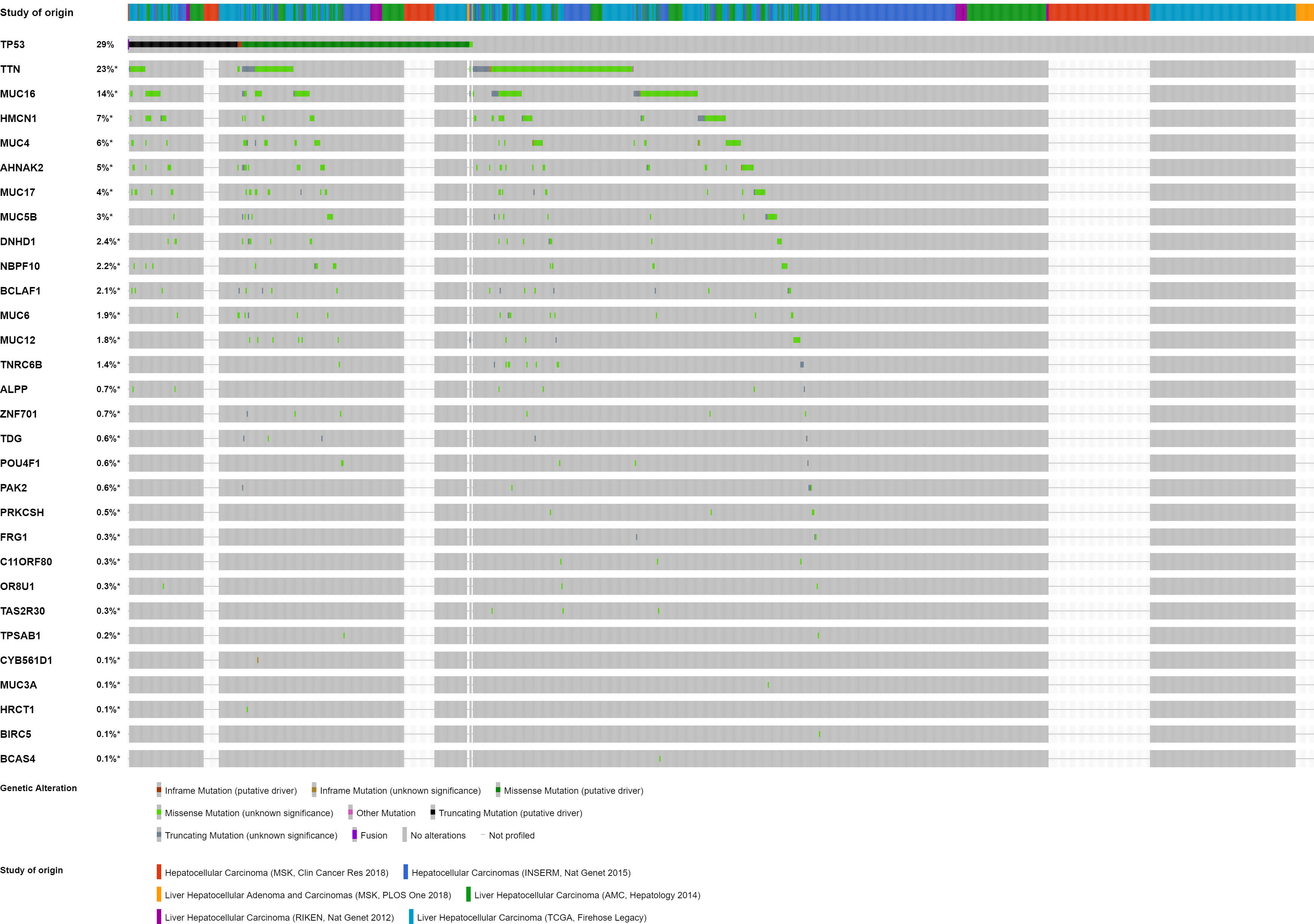

Supplement: Supplementary file 3 [file DataSheet2.ZIP › Supplementary Figures/Supplementary Figure 2.png]

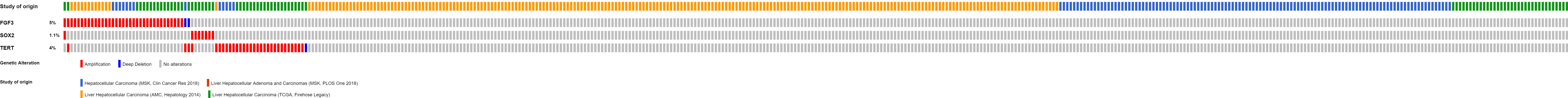

Supplement: Supplementary file 3 [file DataSheet2.ZIP › Supplementary Figures/Supplementary Figure 3.png]

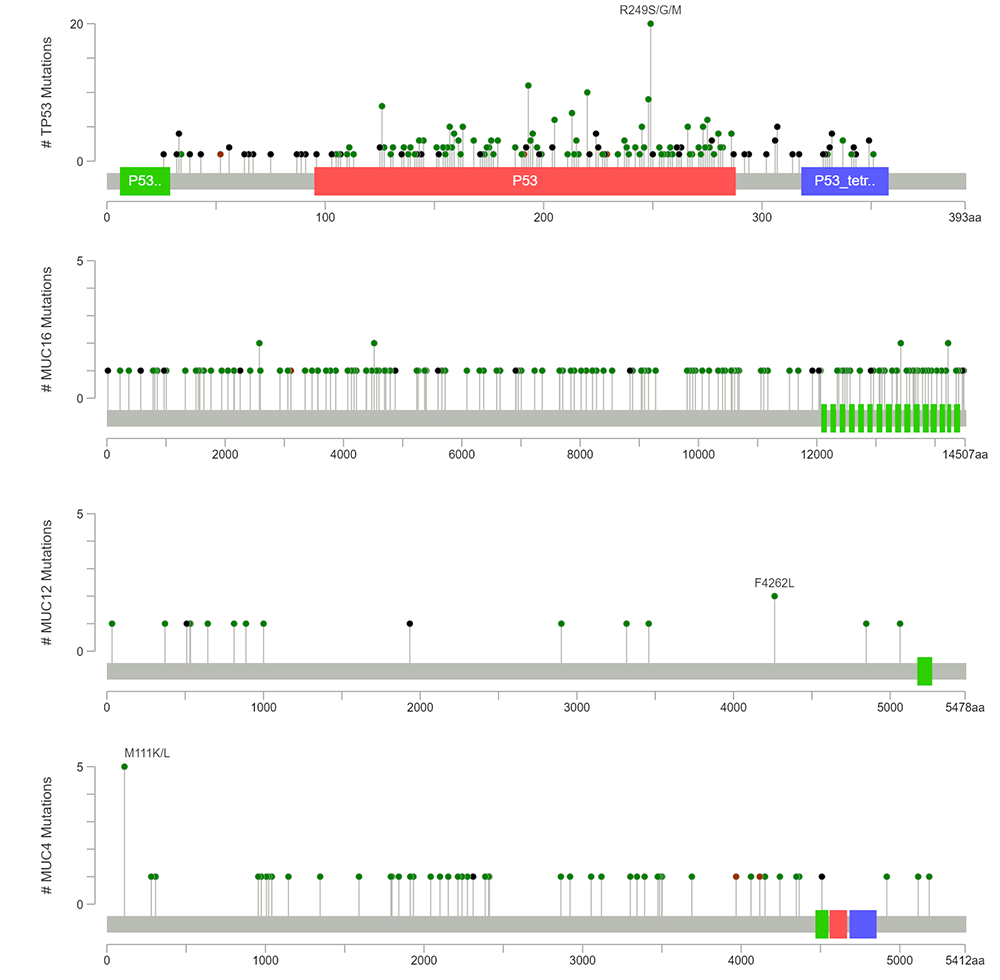

Supplement: Supplementary file 3 [file DataSheet2.ZIP › Supplementary Figures/Supplementary Figure 4.tif]

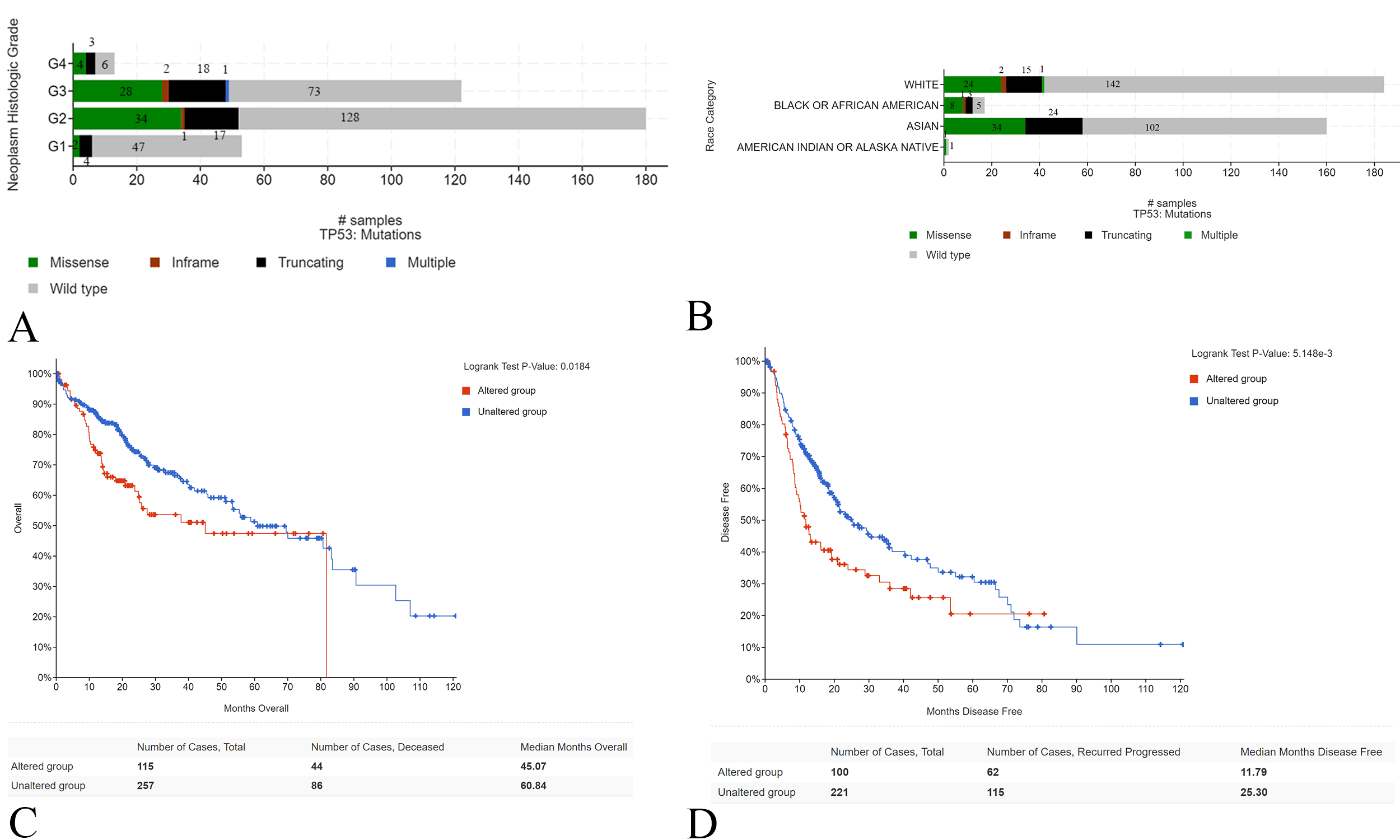

Supplement: Supplementary file 3 [file DataSheet2.ZIP › Supplementary Figures/Supplementary Figure 5.tif]

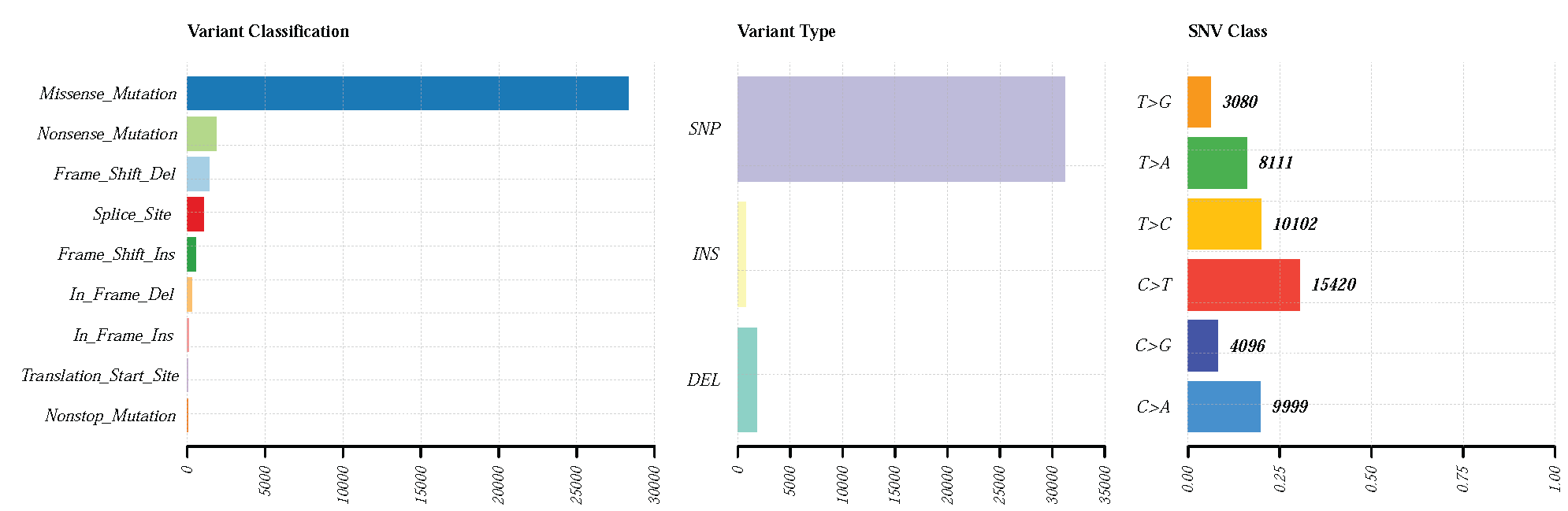

Supplement: Supplementary file 3 [file DataSheet2.ZIP › Supplementary Figures/Supplementary Figure 6.tif]
