# Supplementary material for: Somatic Mutation Profiles Revealed by Next Generation Sequencing (NGS) in 39 Chinese Hepatocellular Carcinoma Patients
Source: Front Mol Biosci. 2022 Jan 18;8:800679. doi: 10.3389/fmolb.2021.800679 (PMC8804344; doi:10.3389/fmolb.2021.800679)
Supplement: Supplementary file 5 [file DataSheet1.docx]

Supplementary Table 1 The clinical characteristics of HCC patients

| Case | Gender | Age | The method of sequencing | HBsAg | HBV-DNA, IU/ml | AFP, ng/ml | Tumor number | The largest tumor diameter, cm | Liver cirrhosis | PVTT | Extrahepatic metastasis | BCLC stage | MVI | Edmondson-Steiner grade | TP53 |
| --- | --- | --- | --- | --- | --- | --- | --- | --- | --- | --- | --- | --- | --- | --- | --- |
| 1 | male | 54 | targeted sequencing | negative | <100 | 1007955 | single | 16.6 | no | no | no | A | yes | II | mutation |
| 2 | male | 47 | targeted sequencing | positive | ≥100 | 311 | single | 12 | no | no | no | A | yes | III | mutation |
| 3 | male | 54 | targeted sequencing | positive | <100 | 58 | multiple | 2.9 | no | no | no | A | yes | II | mutation |
| 4 | male | 44 | targeted sequencing | positive | <100 | 53 | single | 6.2 | no | no | no | A | yes | II | mutation |
| 5 | female | 47 | targeted sequencing | positive | <100 | 68.7 | single | 4 | no | no | no | A | no | II | mutation |
| 6 | male | 65 | targeted sequencing | positive | ≥100 | 6104 | single | 10.1 | no | yes | no | C | no | II | mutation |
| 7 | male | 64 | targeted sequencing | positive | ≥100 | 213285 | single | 8.6 | no | yes | no | C | no | II | mutation |
| 8 | male | 49 | targeted sequencing | positive | ≥100 | 7912.5 | multiple | 7 | no | yes | no | C | no | II-III | wide type |
| 9 | female | 61 | targeted sequencing | positive | <100 | 345 | multiple | 5.2 | no | yes | no | C | no | II | mutation |
| 10 | male | 33 | targeted sequencing | positive | ≥100 | 12 | single | 12.1 | no | yes | no | C | no | III | mutation |
| 11 | male | 48 | targeted sequencing | positive | ≥100 | 61458 | single | 7.1 | no | yes | no | C | no | II | mutation |
| 12 | male | 32 | targeted sequencing | positive | ≥100 | 11588 | single | 8.5 | no | yes | no | C | no | II | wide type |
| 13 | male | 26 | targeted sequencing | positive | <100 | 134744 | multiple | 11.1 | no | yes | Porta hepatis lymph node | C | no | II | mutation |
| 14 | male | 39 | targeted sequencing | negative | <100 | 2.41 | single | 17.3 | no | yes | Diaphragm | C | no | IV | mutation |
| 15 | male | 47 | targeted sequencing | positive | <100 | 2096 | single | 2 | no | no | Abdominal wall | A | no | II-III | wide type |
| 16 | male | 46 | targeted sequencing | positive | ≥100 | 843 | single | 9 | no | no | Left adrenal gland | A | no | II | wide type |
| 17 | male | 38 | targeted sequencing | positive | <100 | 120000 | missing data | missing data | no | no | Abdominal wall | A | no | III | mutation |
| 18 | male | 58 | WES | negative | ≥100 | 453873.56 | multiple | 12.6 | no | yes | no | C | yes | II | mutation |
| 19 | male | 70 | WES | negative | ＜100 | 447.64 | multiple | 10 | no | no | no | B | yes | II | mutation |
| 20 | male | 51 | WES | negative | ＜100 | 36.64 | multiple | 14 | no | no | no | B | yes | II | mutation |
| 21 | male | 49 | WES | negative | ＜100 | 5.9 | multiple | 14 | no | no | no | B | yes | II | wide type |
| 22 | male | 60 | WES | negative | ＜100 | 2.05 | multiple | 15 | yes | no | no | B | no | II | mutation |
| 23 | male | 59 | WES | positive | ＜100 | 457.86 | multiple | 15 | no | no | no | B | no | II | mutation |
| 24 | male | 68 | WES | positive | ≥100 | 7.67 | single | 3 | no | no | no | A | no | II | mutation |
| 25 | male | 37 | WES | positive | ＜100 | 30.65 | single | 3 | no | no | no | A | no | II | mutation |
| 26 | female | 56 | WES | positive | ≥100 | 1724.54 | single | 4 | yes | no | no | A | no | III | mutation |
| 27 | male | 36 | WES | negative | ＜100 | 555.11 | single | 11.8 | no | no | no | A | no | II-III | mutation |
| 28 | male | 46 | WES | positive | ≥100 | >80000 | multiple | 10 | no | no | no | B | yes | II-III | mutation |
| 29 | male | 38 | WES | positive | ≥100 | 44234.78 | multiple | 7 | no | yes | sacrum | C | yes | II-III | mutation |
| 30 | male | 51 | WES | negative | ＜100 | 4.86 | single | 2.5 | no | no | no | A | no | II | mutation |
| 31 | male | 43 | WES | positive | ≥100 | 249.63 | multiple | 5.8 | no | no | spine | C | yes | II-III | wide type |
| 32 | male | 33 | WES | positive | ≥100 | 39 | single | 8.8 | no | no | no | A | yes | II | mutation |
| 33 | male | 60 | WES | positive | ＜100 | 6.25 | single | 4 | yes | no | no | A | yes | II | wide type |
| 34 | male | 66 | WES | positive | ＜100 | 64.54 | single | 3 | yes | no | no | A | yes | II | mutation |
| 35 | male | 38 | WES | positive | ≥100 | 16461.67 | multiple | 22 | no | yes | no | C | no | II | mutation |
| 36 | male | 41 | WES | positive | ≥100 | 4.02 | multiple | 9.9 | no | no | no | B | no | III | mutation |
| 37 | male | 62 | WES | positive | ＜100 | 2.79 | multiple | 11.4 | yes | no | no | B | yes | III | mutation |
| 38 | male | 43 | WES | positive | ＜100 | 4.76 | multiple | 3 | no | no | no | B | yes | III | wide type |
| 39 | male | 46 | WES | negative | ≥100 | 1558450.52 | multiple | 18 | no | yes | no | C | yes | III | wide type |

WES: whole exome sequencing; HBV: hepatitis B virus; PVTT: portal vein tumor thrombus; MVI: microvascular invasion; BCLC: Barcelona clinic liver cancer.

Supplementary Table 2 Summary of gene variations in HCC detected by targeted panel sequencing

| Case | Somatic variation with clinical significance^†^(specific types of variation, variation in abundance /copy number) | Somatic variation with unclear clinical significance | Recommended drugs^‡^ | Variant genes targeted by drugs (signaling pathways) |
| --- | --- | --- | --- | --- |
| 1 | TP53 (V157F, 47.70%) | RB1 (p.T168A) | None | — |
| 2 | TP53 (p.R249S Exon7, 0.44%) | TSC2 (p.E1490G), ARID1A (p.A2235T), BRCA2 (p.D1898G), FGFR3 (p.D580N) | None | — |
| 3 | TP53 (E258*, 25.40%; F270V, 2.00%), TSC1 (R786*, 20.70%) | NOTCH2 (p.N998S), MEN1 (p.R206C), NTRK2 (P.F122C) | Everolimus, Temsirolimus | TSC1 (PI3K/AKT/mTOR) |
| 4 | CCND1 (copy number gain, 16), FGF19 (copy number gain, 10), CTNNB1 (p.D32N, 8.70%), TP53 (p.E258K, 40.30%), FGF4 (copy number gain, 10) | CDKN2B (p.R48S), MSH2 (p.K334E), NTRK3 (p.V289E), KMT2C (p.Y3879F), KRAS (p.V29M), PDCD1LG2 (p.C192G) | Everolimus, Temsirolimus | CCND1 (PI3K/AKT/mTOR) |
| 5 | AMER1 (p.E291*, 53.90%), RB1 (copy number loss, 1), STK24 (copy number gain, 3), CTNNB1 (p.S37F, 23.60%), IRS2 (copy number gain, 3), TP53 (p.R158L, 75.30%) | LCK (p.E288K) | None | — |
| 6 | AXIN1 (H662Mfs*43, 21.30%) | TP53 (p.R174W), MTOR (p.K1293R), FLT1 (p.R598*), BRIP1 (p.Q665L), LRP1B (p.D1096N), ATR (p.T2504N) | None | — |
| 7 | TP53 (c.673-2A>T, 54.9%) | KIT (p.R2S), NF1 (p.I826L), BRCA2 (p.S767C), CDKN2A (p.P64P), CDK12 (p.R722H), KEAP1 (p.Q619Sfs*53), LRP1B (p.Y1865N), BARD1 (p.Q245L) | None | — |
| 8 | NTRK1 (copy number gain, 5), RB1 (p.S393Rfs*8, 74.9%), RIT1 (copy number gain, 6), XIAP (copy number gain, 4), GATA4 (copy number loss, 0), PAK3 (copy number gain, 4), MCL1 (copy number gain, 10) | ALK (p.Q27L), SPTA1 (copy number gain), BLK (copy number loss), STAG2 (copy number gain), EGF (p.G392R), FAT1 (p.G747A), SDHC (copy number gain), BCORL1 (copy number gain), JAK3 (p.T714K), FGFR1 (copy number loss) | Crizotinib | ALK (PI3K/AKT/mTOR, MAPK/ERK, JAK-STAT3) |
| 9 | MET (copy number gain, 20), CDK6 (copy number gain, 8), GRM3 (copy number gain, 8), TP53 (p.V157F, 32.00%) | EGF (p.P644S), PIK3CG (copy number gain), EZH2 (p.T465S) | Crizotinib, Cabozantinib | MET (Ras/Raf/Mek/Erk,  PI3K/AKT/mTOR) |
| 10 | TP53 (R249S), PTEN (c.1154-2A>T), MCL1 (copy number gain) | ASXL1 (p.N1343K), MYCN (p.P75R) | Everolimus, Temsirolimus, | PTEN (PI3K/AKT/mTOR) |
| 11 | TP53 (R249S), MYC (copy number gain), STK11 (D194Y), GNAS (copy number gain) | FGFR4 (p.K169Q), RUNX1T1 (p.A150G) | Everolimus, Temsirolimus | STK11 (PI3K/AKT/mTOR) |
| 12 | AXIN1 (p.W444*, 66.50%), PRDM1 (p.W199*, 34.50%) | CD79A (p.R166S), WEE2 (p.M375K) | None | — |
| 13 | TP53（R249S）, CCDC6-RET（gene fusion）, MYC（copy number gain） | TCF7L2 (c.1201-2A>T) | Sorafenib, Sunitinib, Vandetanib, Cabozantinib, Ponatinib, Regorafenib, Lenvatinib | CCDC6-RET (Ras/Raf/Mek/Erk,  PI3K/AKT/mTOR) |
| 14 | TP53 (L194R) | ZNF217 (p.P823S), MAP2K4 (copy number gain), TSC2 (exon5-exon16 dup) | None | — |
| 15 | PIK3CA (V344M, 1.30%) | KDM5A (p.K138*), SMARCA4 (p.S332F) | None | — |
| 16 | RPTOR (copy number gain, 6), JAK1 (p.S703I exon 15, 46.20%), GNA13 (copy number gain, 6), CEBPA (copy number gain, 4), BIRC5 (copy number gain, 6), IRS2 (copy number gain, 3), SOX9 (copy number gain, 6) | CD79B (copy number gain), KMT2D (p.G5181V), PRKAR1A (copy number gain), NTRK3 (p.V550I), CTNNA1 (p.L61F), ABL2 (p.G39W), ZNF750 (copy number gain) | Everolimus, Temsirolimus, Ruxolitinib | RPTOR (PI3K/AKT/mTOR)、JAK1 (JAK-STAT) |
| 17 | TP53 (Q136Hfs*34), NOTCH2 (copy number gain), HSD3B1 (copy number gain) | TEK (p.T922M), NKX2-1 (p.Q182Nfs*16) | None | — |

†: 1) NCCN guidelines recommend these genes to be tested. 2) Genes correspond some therapeutic drugs with clinical or preclinical evidence, but have not been included in the guidelines.

‡: Recommend drugs: Drugs approved by the FDA/CFDA (China Food and Drug Administration) for HCC or other tumors.

Supplementary Table 4 Summary of key gene variations detected by whole-exome sequencing

| Case | TMB, Muts/MB | Immunotherapy expectation ORR (%) | MSI (%) | CNV (%) | Significant tumor driver genes variation that may have clinical significance (specific types of variation, variation frequency/copy number) | Important tumor driver genes with unknown function of variation site or no corresponding targeted drugs (specific types of variation, variation frequency/copy number) | Variant genes targeted by marketing targeted drugs (signal pathway/molecular mechanism) |
| --- | --- | --- | --- | --- | --- | --- | --- |
| 18 | 9.10 | 23.15 | 0.16 | 2.22 | BRCA1 (copy number loss, 1.16), FGF3 (copy number gain, 3.02), NRAS (p.G13R, 51.06%), TP53 (p.R337L, 58.06%) | SOX2 (copy number gain, 3.30), AXIN1 (p.R284fs, 58.82%) | BRCA1 (BRCA1/2), FGF3 (FGFR), NRAS (RAS/RAF/MEK) |
| 19 | 8.89 | 22.90 | 0.00 | 0.76 | TP53 (p.P151S, 12.35%) | GNAQ (p.T96S, 6.67%), JAK2 (p.V341G, 18.75%), TSC2 (p.G1204V, 10.00%) | None |
| 20 | 8.65 | 22.60 | 0.05 | 1.21 | TP53 (p.R249S, 41.82%) | ALK (p.R1214H, 6.82%), EWSR1 (p.Y226C, 33.33%), GNA11 (p.A108_N109del, 22.73%), HNF1A (p.G554fs, 52.54%), MCL1 (p.T280_I281>SV, 7.58%), RPTOR (p.A1258S, 22.26%), SOX2 (copy number gain) | None |
| 21 | 8.29 | 22.14 | 0.03 | 0.58 | FGFR3 (copy number gain, 4.2), JAK1 (p.S703I, 13.64%) | CREBBP (p.N522I, 15.00%), ERBB2 (p.G464*, 12.13%), GNAQ (p.T96S, 18.18%) | FGFR3 (FGFR), AK1 (JAK/STAT) |
| 22 | 8.26 | 22.10 | 0.88 | 8.16 | ATR (p.A971fs, 61.45%), TP53 (p.R249S, 66.20%) | ALK (p.R1212S, 20.48%), RAF1 (p.N553Y, 38.57%), SMARCA4 (splice site c.4912-2A>C, 8.03%), TERT (copy number gain, 3.23) | ATR (DNA mismatch repair) |
| 23 | 8.23 | 22.06 | 0.00 | 1.01 | TP53 (p.R273C, 41.18%) | FGFR2 (p.G488R, 8.89%), ROS1 (p.S720G, 27.27%), SOX2 (copy number gain, 3.23) | None |
| 24 | 7.63 | 21.25 | 0.34 | 1.74 | CCND1 (copy number gain, 6.6), FGF19/FGF3/FGF4 (copy number gain, 6.6), APC (copy number loss, 1.18), TP53 (p.R213*, 62.92%) | CARD11 (p.L780Q, 38.72%), HNF1A (p.M1?, 40.56%), KMT2C (p.R909K, 6.25%; p.D348N, 6.45%) | CCND1 (cell cycle), FGF19/FGF3/FGF4 (FGFR) |
| 25 | 7.39 | 20.90 | 0.00 | 0.48 | TP53 (p.H179Y, 10.07%) | — | None |
| 26 | 7.33 | 20.81 | 0.05 | 1.17 | AKT1 (copy number gain, 3.01), FGF3 (copy number gain, 3.01), FGFR3 (copy number gain, 3.4), CTNNB1 (p.S45P, 31.82%), TP53 (p.H178P, 52.87%), | APC (p.M743I, 37.33%) | AKT1 (PI3K/AKT/mTOR), FGF3/FGFR3 (FGFR) |
| 27 | 6.63 | 19.73 | 0.53 | 0.86 | CCND3 (copy number gain, 3.02), GNAS (p.R201C, 20.03%), CTNNB1 (p.D32H, 18.14%), TP53 (p.G105S, 65.49%) | AXL (splice site c.1900-2A>G, 6.57%), ERBB2 (p.P1130L, 16.67%), FOXL2 (p.L195Q, 14.29%). HIST1H3B (p.G45A, 42.73%), KMT2A (p.T3543I, 30.77%), NOTCH1 (p.G1157R, 6.17%), TERT (copy number gain, 3.00) | CCND3 (cell cycle), GNAS (G-protein) |
| 28 | 6.46 | 19.44 | 25.00 | 1.48 | TP53 (p.G226fs, 25.63%), CCND1 (copy number gain, 4.73), FGF19 (copy number gain, 3.98), FGF3 (copy number gain, 4.91), FGF4 (copy number gain, 3.95) | CDKN2A (p.G89C, 33.99%), CIC (p.R2108Q, 16.83%), FGFR1 (p.H810Q, 6.90%), NOTCH2 (p.H107P, 13.65%), SOX2 (copy number gain, 3.62), TERT (copy number gain, 3.60) | BRCA2 (BRCA1/2), CCND1 (cell cycle), FGF3/FGF4/FGF19 (FGFR) |
| 29 | 6.37 | 19.30 | 17.00 | 1.21 | TP53 (p.R249W, 44.63%)、APC (p.R1331fs, 47.62%) | AR (p.R787Q, 69.57%、NF1 (p.T1883I, 28.85%) | None |
| 30 | 6.16 | 18.94 | 0.00 | 0.96 | TP53 (p.C176Y, 8.01%) | BTK (p.V535A, 6.15%)、MLH3 (p.D1131Y, 6.56%) | None |
| 31 | 5.86 | 18.39 | 0.10 | 0.16 | None | ATRX (p.H180R, 38.10%), RET (p.L206H, 5.68%) | None |
| 32 | 5.65 | 18.00 | 0.02 | 4.68 | TSC2 (p.E1442fs, 24.76%), TP53 (p.C176W, 22.13%) | CSF3R (p.A205V, 12.42%), RB1 (p.S567*, 17.65%) | TSC2 (TSC1/2) |
| 33 | 5.59 | 17.89 | 0.00 | 1.65 | FGF3 (copy number gain, 3.33) | SOX2 (copy number gain, 3.37) | FGF3 (FGFR) |
| 34 | 5.43 | 17.57 | 0.86 | 11.49 | ATK3 (copy number gain, 3.16), FGF3 (copy number gain, 3.06), ARID1A (splice site c.3539+1G>A, 50.21%), TP53 (p.R337C, 45.37%) | B2M (copy nimber loss, 0.71), EPCAM (p.E85D, 17.89%), GNAQ (p.T187A, 45.28%), SUFU (p.G11R, 17.65%), TERT (copy number gain, 3.91) | AKT3 (PI3K/Akt/mTOR), FGF3 (FGFR) |
| 35 | 4.94 | 16.56 | 0.07 | 1.76 | NRAS (p.G12D, 1.05%), TP53 (p.S215G, 17.48%) | BRCA1 (p.E1339K, 15.38%), NOTCH2 (p.A3F, 30.77%), TERT (copy number gain, 3.07) | NRAS (RAS/RAF/MEK) |
| 36 | 4.71 | 16.05 | 0.04 | 1.52 | BRCA2 (copy number loss, 1.15), PIK3CA (p.E1037K, 10.03%), PTEN (p.G165E, 24.55%), PTEN (p.M264I, 12.83%), TP53 (p.R249S, 60.49%), TP53 (p.R273H, 28.18%) | B2M (p.M119fs, 19.35%), KMT2C (p.R894Q, 8.82%), MSH3 (p.S220L, 27.78%), POLE (p.H342P, 10.10%), RET (p.I880V, 40.00%) | BRCA2 (BRCA1/2), PIK3CA/PTEN (PI3K/Akt/mTOR) |
| 37 | 3.91 | 14.04 | 6.00 | 4.41 | JAK1 (p.S703I, 11.63%), TSC2 (splice site c.2355+2T>C, 20.63%), TP53 (p.G105V, 8.85%) | None | JAK1 (JAK/STAT), TSC2 (TSC1/2) |
| 38 | 3.40 | 12.52 | 0.29 | 19.56 | ERBB2 (copy number gain, 3.04) | ARID1A (p.D1963E, 11.12%), MCL1 (copy number gain, 3.88) | ERBB2 (ErbB family) |
| 39 | 3.03 | 11.27 | 33 | 1.32 | MSH6 (p.A35fs, 51.05%)、PTEN (p.Q245*, 1.44%)、CTNNB1 (p.T41A, 51.38%) | ATM (p.R337H, 1.75%)、EP300 (p.C364R, 38.04%) 、NOTCH1 (p.D352N, 1.62%) | MSH6 (DNA mismatch repair), PTEN (PI3K/AKT/mTOR) |

TMB: tumor mutation burden; ORR: objective response rate; MSI: microsatellite instability; CNV: copy number variation.

Supplementary Table 5 Summary of top 4 frequent gene mutation detected by whole-exome sequencing

| Gene | Case | Nucleotide alteration | Amino acid alteration | Mutation frequency | Mutation type | Mutation taster prediction |
| --- | --- | --- | --- | --- | --- | --- |
| TP53 | 18 | c.1010G>T | p.R337L | 58.06% | missense | Deleterious |
|  | 19 | c.451C>T | p.P151S | 12.35% | missense | Deleterious |
|  | 20 | c.747G>T | p.R249S | 41.82% | missense | Deleterious |
|  | 22 | c.747G>T | p.R249S | 66.20% | missense | Deleterious |
|  | 23 | c.817C>T | p.R273C | 41.18% | missense | Deleterious |
|  | 24 | c.637C>T | p.R213* | 62.92% | nonsense | Deleterious |
|  | 25 | c.535C>T | p.H179Y | 10.07% | missense | Deleterious |
|  | 26 | c.533A>C | p.H178P | 52.87% | missense | Deleterious |
|  | 27 | c.313G>A | p.G105S | 65.49% | missense | Deleterious |
|  | 28 | c.675delT | p.G226fs | 25.63% | frameshift mutation | Deleterious |
|  | 29 | c.745A>T | p.R249W | 44.63% | missense | Deleterious |
|  | 30 | c.527G>A | p.C176Y | 8.01% | missense | Deleterious |
|  | 32 | c.528C>G | p.C176W | 22.13% | missense | Deleterious |
|  | 34 | c.1009C>T | p.R337C | 45.37% | missense | Deleterious |
|  | 35 | c.643A>G | p.S215G | 17.48% | missense | Deleterious |
|  | 36 | c.747G>T | p.R249S | 60.49% | missense | Deleterious |
|  | 36 | c.818G>A | p.R273H | 28.18% | missense | Deleterious |
|  | 37 | c.314G>T | p.G105V | 8.85% | missense | Deleterious |
| MUC16 | 18 | c.40219G>A | p.D13407N | 11.76% | missense | unknown |
|  | 19 | c.40625G>A | p.G13542E | 8.45% | missense | unknown |
|  | 20 | c.40297C>G | p.H13433D | 17.65% | missense | unknown |
|  | 20 | c.40312A>G | p.N13438D | 20.00% | missense | unknown |
|  | 20 | c.38206G>A | p.G12736S | 5.63% | missense | unknown |
|  | 20 | c.40907T>C | p.L13636P | 5.26% | missense | unknown |
|  | 20 | c.38214G>T | p.M12738I | 5.80% | missense | unknown |
|  | 20 | c.37570_37571delCT | p.L12524fs | 6.82% | frameshift mutation | unknown |
|  | 20 | c.37567G>T | p.D12523Y | 7.14% | missense | unknown |
|  | 20 | c.38224A>T | p.T12742S | 6.35% | missense | unknown |
|  | 25 | c.39086G>A | p.R13029H | 5.48% | missense | unknown |
|  | 25 | c.39089A>G | p.H13030R | 5.63% | missense | unknown |
|  | 26 | c.37205G>A | p.G12402E | 6.52% | missense | unknown |
|  | 28 | c.40820T>C | p.I13607T | 8.25% | missense | unknown |
|  | 32 | c.40661G>A | p.R13554H | 6.35% | missense | unknown |
|  | 32 | c.40666G>A | p.D13556N | 6.40% | missense | unknown |
|  | 32 | c.40677C>A | p.S13559R | 6.45% | missense | unknown |
|  | 32 | c.40754C>G | p.T13585S | 11.76% | missense | unknown |
|  | 34 | c.29942C>T | p.A9981V | 47.67% | missense | unknown |
|  | 34 | c.31047T>G | p.H10349Q | 11.89% | missense | unknown |
|  | 35 | c.39221G>A | p.G13074E | 5.98% | missense | unknown |
|  | 35 | c.39220G>A | p.G13074R | 5.22% | missense | unknown |
|  | 35 | c.39232G>A | p.G13078R | 6.25% | missense | unknown |
|  | 35 | c.39260T>C | p.L13087P | 5.16% | missense | unknown |
|  | 35 | c.39262A>G | p.N13088D | 5.13% | missense | unknown |
|  | 35 | c.39208T>C | p.S13070P | 5.05% | missense | unknown |
|  | 36 | c.39790C>A | p.H13264N | 14.44% | missense | unknown |
|  | 36 | c.39761A>G | p.Q13254R | 6.03% | missense | unknown |
| MUC12 | 20 | c.276T>A | p.S92R | 10.81% | missense | unknown |
|  | 20 | c.283G>A | p.V95M | 11.11% | missense | unknown |
|  | 20 | c.299G>A | p.W100* | 12.12% | nonsense | unknown |
|  | 20 | c.301A>G | p.M101V | 14.71% | missense | unknown |
|  | 20 | c.307G>A | p.G103R | 12.50% | missense | unknown |
|  | 20 | c.9877G>A | p.G3293S | 6.52% | missense | unknown |
|  | 20 | c.273G>C | p.E91D | 12.82% | missense | unknown |
|  | 21 | c.5069A>T | p.K1690I | 24.00% | missense | unknown |
|  | 22 | c.2120C>T | p.S707F | 18.18% | missense | unknown |
|  | 23 | c.1348A>G | p.S450G | 6.98% | missense | unknown |
|  | 23 | c.1652C>A | p.A551E | 5.75% | missense | unknown |
|  | 24 | c.12599G>A | p.G4200D | 9.23% | missense | unknown |
|  | 25 | c.6112G>T | p.G2038C | 18.75% | missense | unknown |
|  | 28 | c.7287_7293delCGCCAGG | p.P2430fs | 7.89% | frameshift mutation | unknown |
|  | 29 | c.13106T>C | p.M4369T | 19.35% | missense | unknown |
|  | 33 | c.242C>G | p.S81C | 9.09% | missense | unknown |
|  | 33 | c.13106T>C | p.M4369T | 12.00% | missense | unknown |
|  | 35 | c.3376G>A | p.G1126S | 13.04% | missense | unknown |
|  | 35 | c.3368T>C | p.L1123P | 12.86% | missense | unknown |
| MUC4 | 18 | c.5278C>T | p.P1760S | 6.67% | missense | unknown |
|  | 20 | c.11365G>C | p.D3789H | 13.89% | missense | unknown |
|  | 20 | c.11374C>T | p.P3792S | 11.43% | missense | unknown |
|  | 20 | c.11387C>G | p.T3796S | 10.00% | missense | unknown |
|  | 20 | c.8975T>C | p.L2992P | 13.64% | missense | unknown |
|  | 22 | c.11498C>T | p.A3833V | 7.46% | missense | unknown |
|  | 22 | c.10526G>A | p.G3509D | 8.06% | missense | unknown |
|  | 22 | c.11368_11369insTG | p.T3790fs | 5.19% | frameshift mutation | unknown |
|  | 22 | c.12067T>A | p.S4023T | 30.77% | missense | unknown |
|  | 22 | c.12080C>A | p.T4027K | 28.57% | missense | unknown |
|  | 22 | c.12085C>G | p.H4029D | 37.50% | missense | unknown |
|  | 22 | c.12088G>A | p.A4030T | 41.18% | missense | unknown |
|  | 22 | c.12097G>C | p.V4033L | 40.00% | missense | unknown |
|  | 22 | c.11477C>T | p.P3826L | 8.06% | missense | unknown |
|  | 23 | c.314C>A | p.P105Q | 26.19% | missense | unknown |
|  | 26 | c.11320G>A | p.A3774T | 12.00% | missense | unknown |
|  | 26 | c.6286T>C | p.S2096P | 7.22% | missense | unknown |
|  | 26 | c.4639A>T | p.T1547S | 5.36% | missense | unknown |
|  | 26 | c.11317C>G | p.H3773D | 12.00% | missense | unknown |
|  | 26 | c.4645G>C | p.D1549H | 5.36% | missense | unknown |
|  | 27 | c.13945A>G | p.S4649G | 21.83% | missense | unknown |
|  | 32 | c.8810T>C | p.V2937A | 6.82% | missense | unknown |
|  | 32 | c.4727C>T | p.S1576L | 6.10% | missense | unknown |
|  | 32 | c.12521G>C | p.G4174A | 7.69% | missense | unknown |
|  | 33 | c.4648A>G | p.T1550A | 7.89% | missense | unknown |
|  | 33 | c.4727C>T | p.S1576L | 5.00% | missense | unknown |
|  | 33 | c.4657_4690delCTTCCTGTCACCGACGCTTCCTCAGTATCCACAG | p.L1553fs | 5.06% | frameshift mutation | unknown |
|  | 34 | c.12112A>C | p.T4038P | 7.58% | missense | unknown |
|  | 36 | c.629A>C | p.Q210P | 9.66% | missense | unknown |


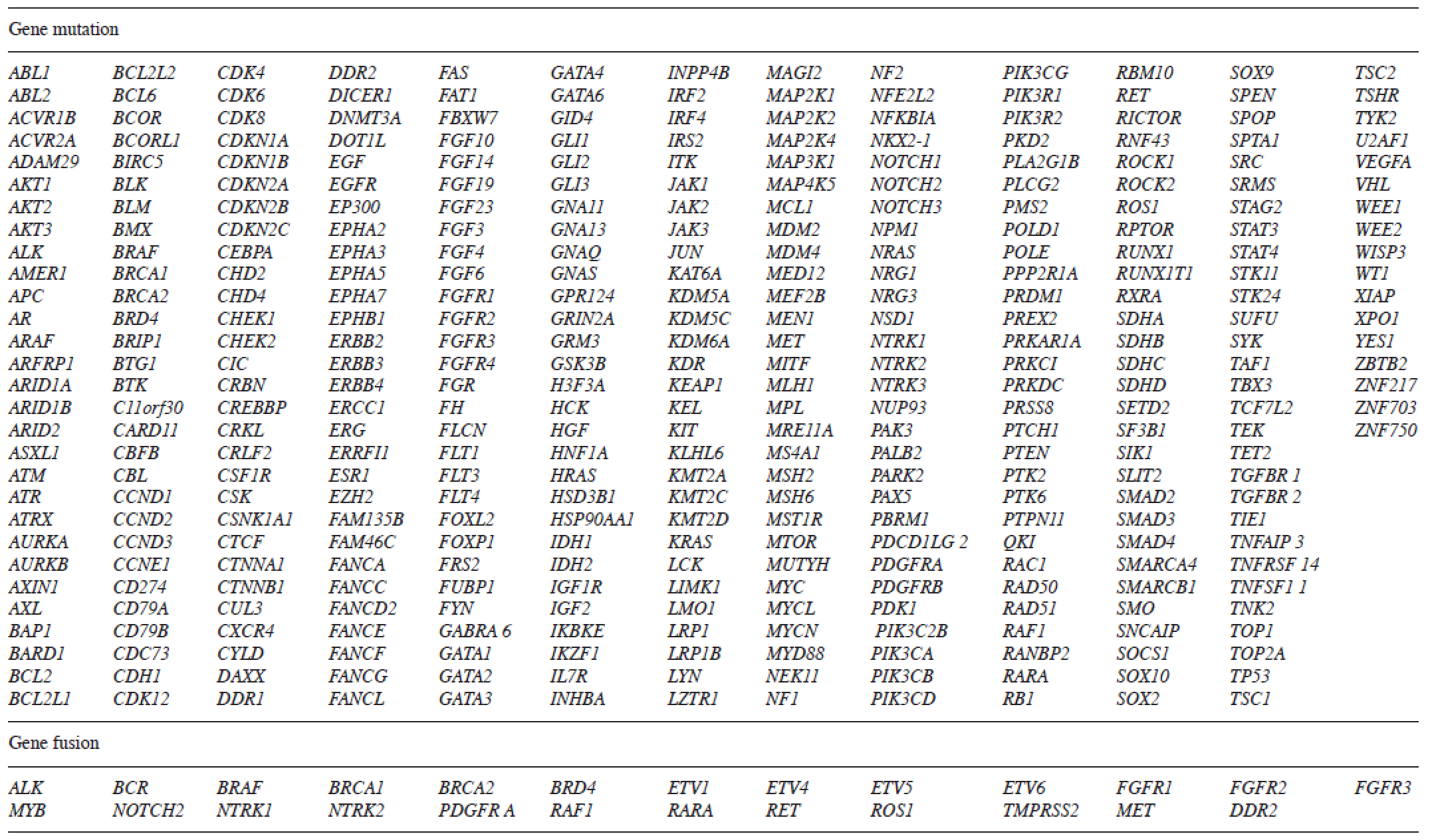


Supplementary Figure 1. Gene list of the targeted panel sequencing.


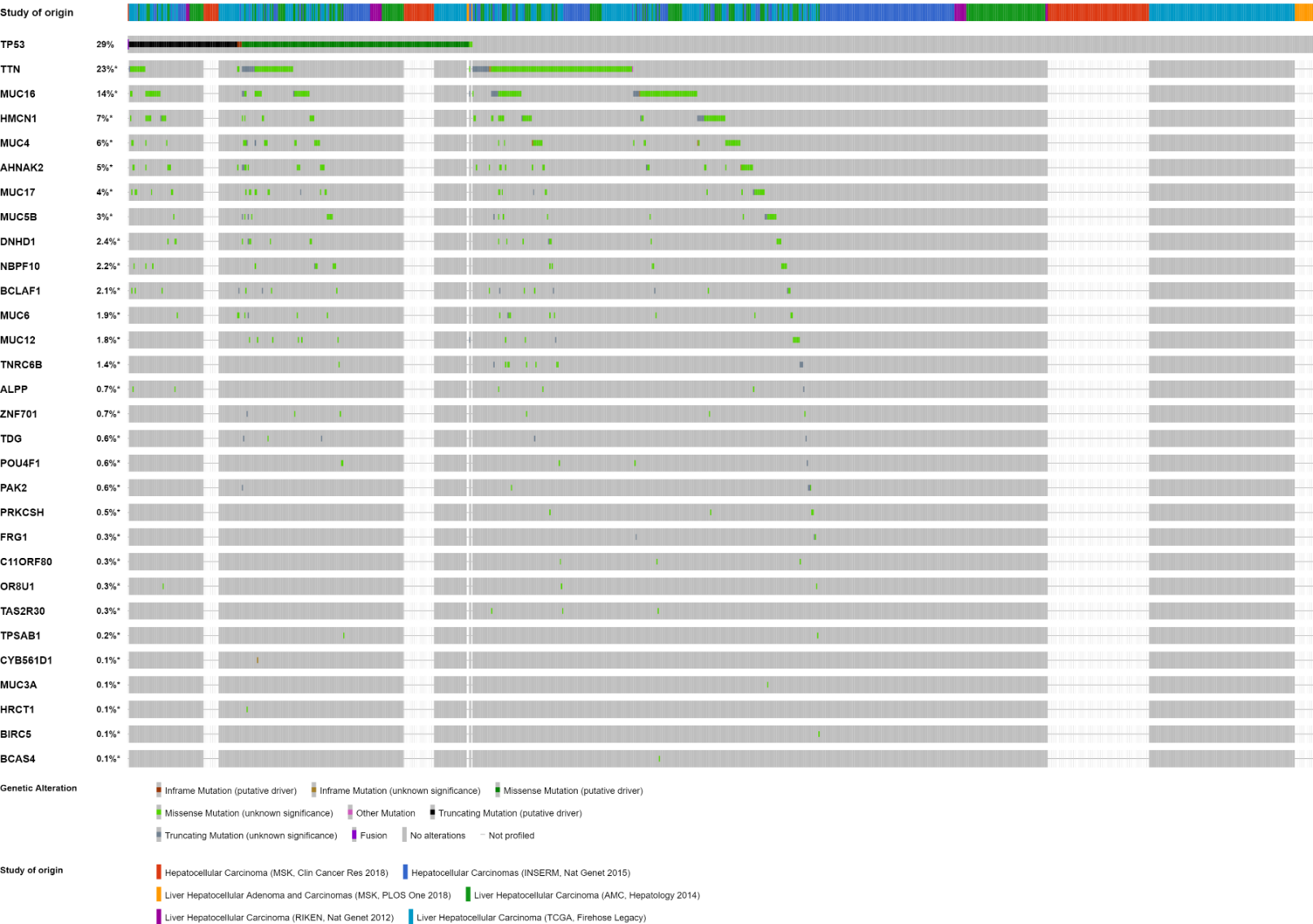


Supplementary Figure 2 OncoPrint of high frequency gene mutation in our cohort verified using cBioPortal. The different types of gene mutation were highlighted in different colors


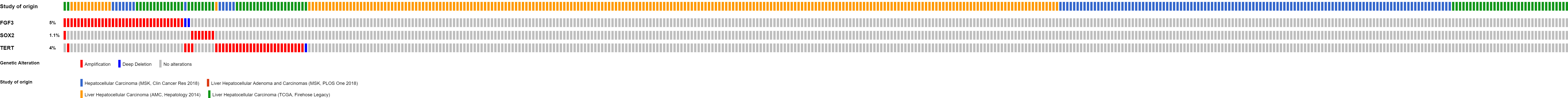


Supplementary Figure 3 OncoPrint of high frequency gene CNV in our cohort verified using cBioPortal. The different types of CNV were highlighted in different colors


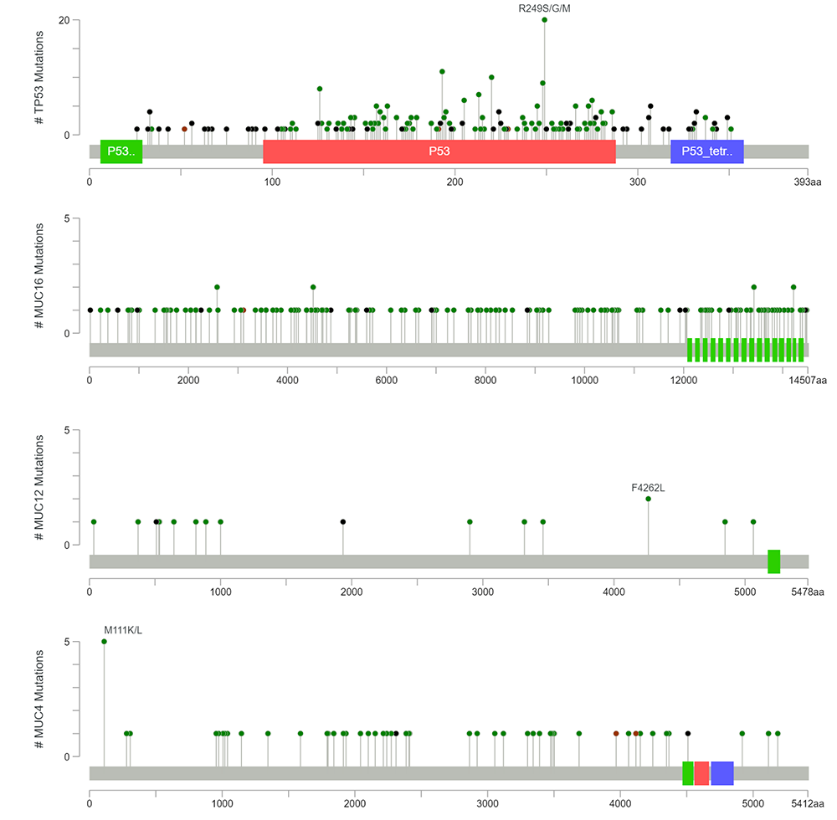


Supplementary Figure 4 Lollipop plot of top 4 mutated genes in our cohort verified using Mutation Mapper (cBioPortal).

green represents missense mutation; black represents truncating mutation (nonsense, nonstop, frameshift deletion, frameshift insertion and splice site); brown represents in-frame mutation (in-frame deletion and in-frame insertion).

TP53: green represents P53_TAD: P53 transactivation motif (6 - 29) PFAM; red represents P53: P53 DNA-binding domain (95 - 288) PFAMMutation Aligner; purple represents P53_tetramer: P53 tetramerisation motif (318 - 358) PFAM Mutation Aligner.

MUC16: green represents SEA: SEA domain (12699 - 12797) PFAM Mutation Aligner.

MUC12: green represents SEA: SEA domain (5173 - 5268) PFAM Mutation Aligner

MUC4：green represents NIDO: Nidogen-like (4468 - 4551) PFAM Mutation Aligner; red represents AMOP: AMOP domain (4557 - 4668) PFAM; purple represents VWD: von Willebrand factor type D domain (4683 - 4854) PFAM Mutation Aligner.


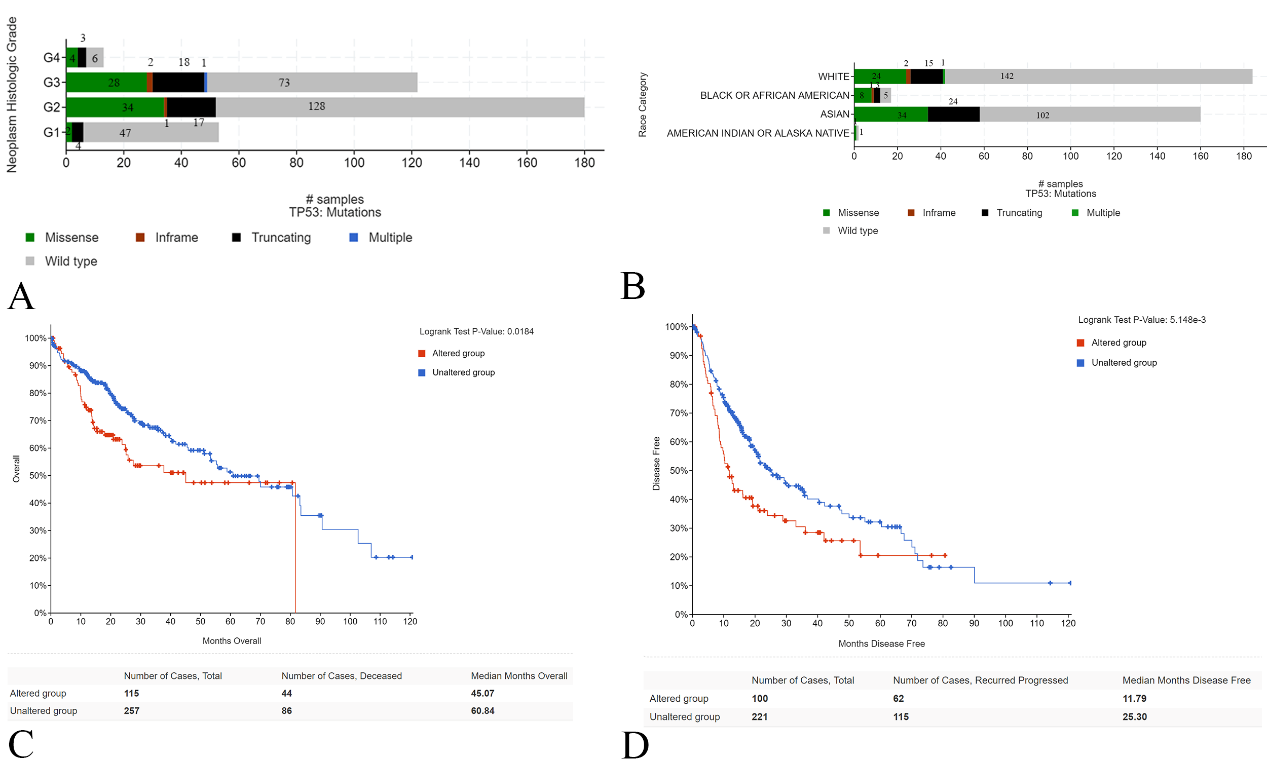


Supplementary Figure 5 The correlation analyses between TP53 mutation and clinical characteristics in cBioPortal. Distribution of TP53 mutation specific types in different histologic grade (A) and race category subgroups (B). TP53 mutation affected OS (C) and DFS (D) in HCC. Altered group means cases with TP53 mutation and unaltered group means cases without TP53 mutation.


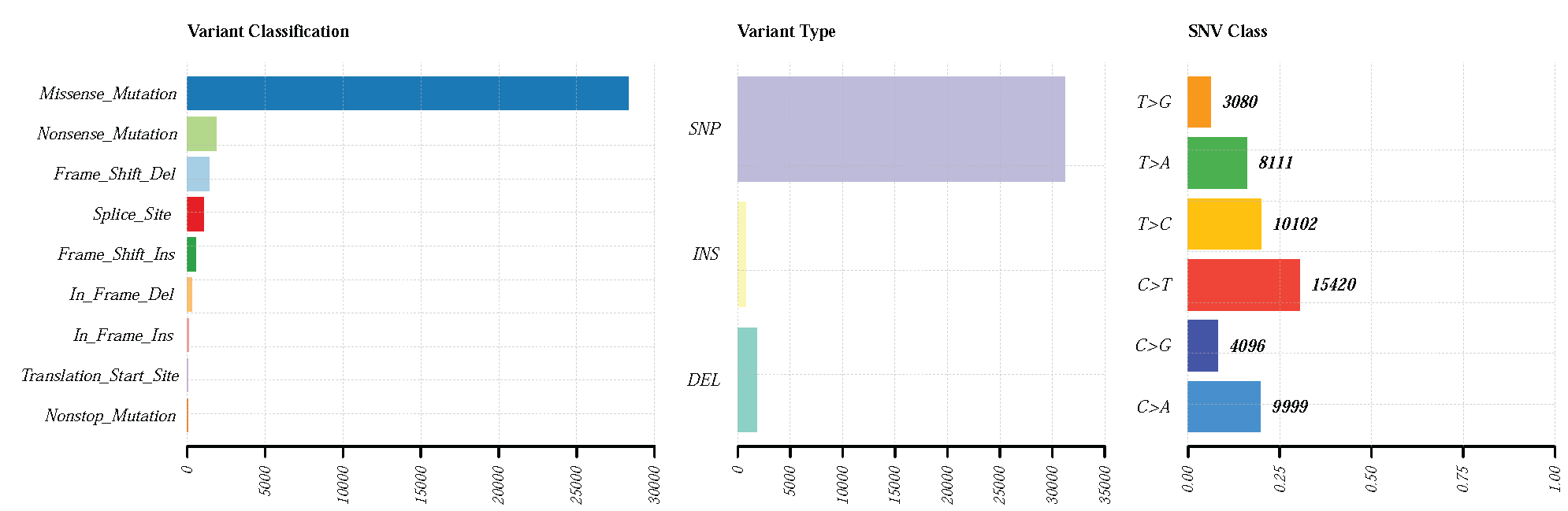


Supplementary Figure 6. Bar chart of the number of nine different types of variation (left), three main types of variation (medium) and 6 types of SNV (right) in TCGA-HCC samples. (The analysis was conducted with the online bioinformatic platform Assistant for Clinical Bioinformatics, https://www.aclbi.com/static/index.html#/)
